# Cell-targeted PD-1 agonists that mimic PD-L1 are potent T cell inhibitors

**DOI:** 10.1172/jci.insight.152468

**Published:** 2021-10-22

**Authors:** Adam P. Curnock, Giovanna Bossi, Jyothi Kumaran, Lindsay J. Bawden, Rita Figueiredo, Rajeevkumar Tawar, Katherine Wiseman, Emma Henderson, Sec Julie Hoong, Veronica Gonzalez, Hemza Ghadbane, David E.O. Knight, Ronan O’Dwyer, David X. Overton, Christina M. Lucato, Nicola M.G. Smith, Carlos R. Reis, Keith Page, Lorraine M. Whaley, Michelle L. McCully, Stephen Hearty, Tara M. Mahon, Peter Weber

**Affiliations:** Immunocore Ltd., Milton Park, Abingdon, United Kingdom.

**Keywords:** Autoimmunity, Immunology, Adaptive immunity, Autoimmune diseases, Drug therapy

## Abstract

The PD-1/PD-L1 pathway is a key immune checkpoint that regulates T cell activation. There is strong rationale to develop PD-1 agonists as therapeutics against autoimmunity, but progress in this area has been limited. Here, we generated T cell receptor (TCR) targeting, PD-1 agonist bispecifics called ImmTAAI molecules that mimic the ability of PD-L1 to facilitate the colocalization of PD-1 with the TCR complex at the target cell–T cell interface. PD-1 agonist ImmTAAI molecules specifically bound to target cells and were highly effective in activating the PD-1 receptor on interacting T cells to achieve immune suppression. Potent PD-1 antibody ImmTAAI molecules closely mimicked the mechanism of action of endogenously expressed PD-L1 in their localization to the target cell–T cell interface, inhibition of proximal TCR signaling events, and suppression of T cell function. At picomolar concentrations, these bispecifics suppressed cytokine production and inhibited CD8^+^ T cell–mediated cytotoxicity in vitro. Crucially, in soluble form, the PD-1 ImmTAAI molecules were inactive and, hence, could avoid systemic immunosuppression. This study outlines a promising new route to generate more effective, potent, tissue-targeted PD-1 agonists that can inhibit T cell function locally with the potential to treat autoimmune and chronic inflammatory diseases of high unmet need.

## Graphical abstract

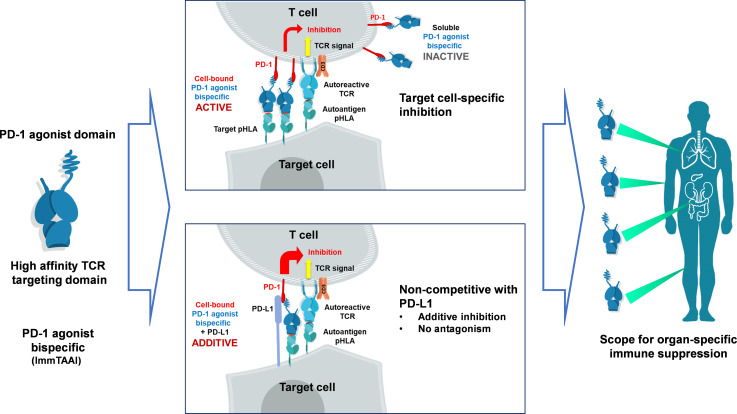

## Introduction

The PD-1 pathway is a key immune checkpoint that plays an important role in maintaining peripheral T cell tolerance and regulating adaptive immune responses (1–3). The PD-1 receptor can be induced on activated T cells, while its natural ligands, PD-L1 and PD-L2, are expressed by various immune and nonimmune cells (4–7). PD-1 and PD-L1 are major targets for therapeutic intervention, particularly in oncology, where several antagonistic PD-1/PD-L1 antibodies that block PD-1–mediated T cell inhibition have demonstrated efficacy in a variety of tumour types and are now approved anticancer therapies (2, 3, 8).

In autoimmunity, there is mounting evidence that PD-1 pathway impairment plays an important role in disease pathogenesis. PD-1, PD-L1, and PD-L2 gene polymorphisms are associated with several autoimmune diseases (9–13), and perturbance of PD-1 pathway components has been reported in some indications. For example, abnormally low PD-L1 expression was noted in samples from type 1 diabetes and Crohn’s disease patients (14). Additionally, PD-1 expression on effector T cells is elevated in a number of autoimmune diseases (15–17), and administration of PD-1 pathway antagonists can cause autoimmune-like symptoms in cancer patients, including aggravation of preexisting autoimmunity (18, 19). Together, these findings suggest that activating PD-1 on autoreactive lymphocytes may serve as a mechanism to treat autoimmune diseases. However, despite strong rationale, few PD-1 agonists have reached the clinic, and efficacy in patients is yet to be demonstrated.

One promising approach to generate efficacious PD-1 agonists is to mimic natural PD-L1–PD-1 engagement at the target cell–T cell interface. PD-L1–mediated activation of PD-1 requires accumulation of the ligand at the cell-cell interface, promoting the colocalization of PD-1 and the T cell receptor (TCR) complex at the immune synapse (20). Ligation of PD-1 enables the recruitment and activation of the SHP2 phosphatase, which in turn dephosphorylates TCR complex–signaling molecules, including ZAP-70, SLP-76, and PLCγ, resulting in T cell inhibition (21–27).

A target cell–bound PD-1 agonist — e.g., localized to pancreatic β cells of a type 1 diabetes patient — could replicate this mechanism to inhibit attacking autoreactive T cells. Such an agent would only activate the PD-1 receptor on directly interacting PD-1^+^ T cells and trigger localized immune suppression without impairment of the immune response in the periphery. To mimic natural PD-L1–PD-1 engagement, we engineered bispecific PD-1 agonists that can specifically bind to target cells and simultaneously activate PD-1 on interacting T cells. To do so, recombinant PD-L1 or anti–PD-1 agonist antibodies were fused to a recombinant, affinity-enhanced TCR with picomolar affinity against a target cell peptide–HLA complex. These targeted bispecifics, hereafter referred to as PD-1 agonist ImmTAAI (immune modulating monoclonal TCR against autoimmunity) molecules, effectively act as a bridge, ensuring PD-1 agonism occurs in cis with TCR engagement on the attacking autoreactive T cell.

In this study, we investigated whether target cell binding can increase the ability of PD-1 agonists to inhibit T cell signaling and function. We found that targeted PD-1 agonist ImmTAAI molecules are potent inhibitors of effector T cells, offering a promising route to successfully exploit this pathway and generate target cell–specific T cell inhibitors for the treatment of autoimmune diseases.

## Results

### Target cell–bound PD-L1 ImmTAAI molecules inhibit TCR signaling.

**Figure 1:**
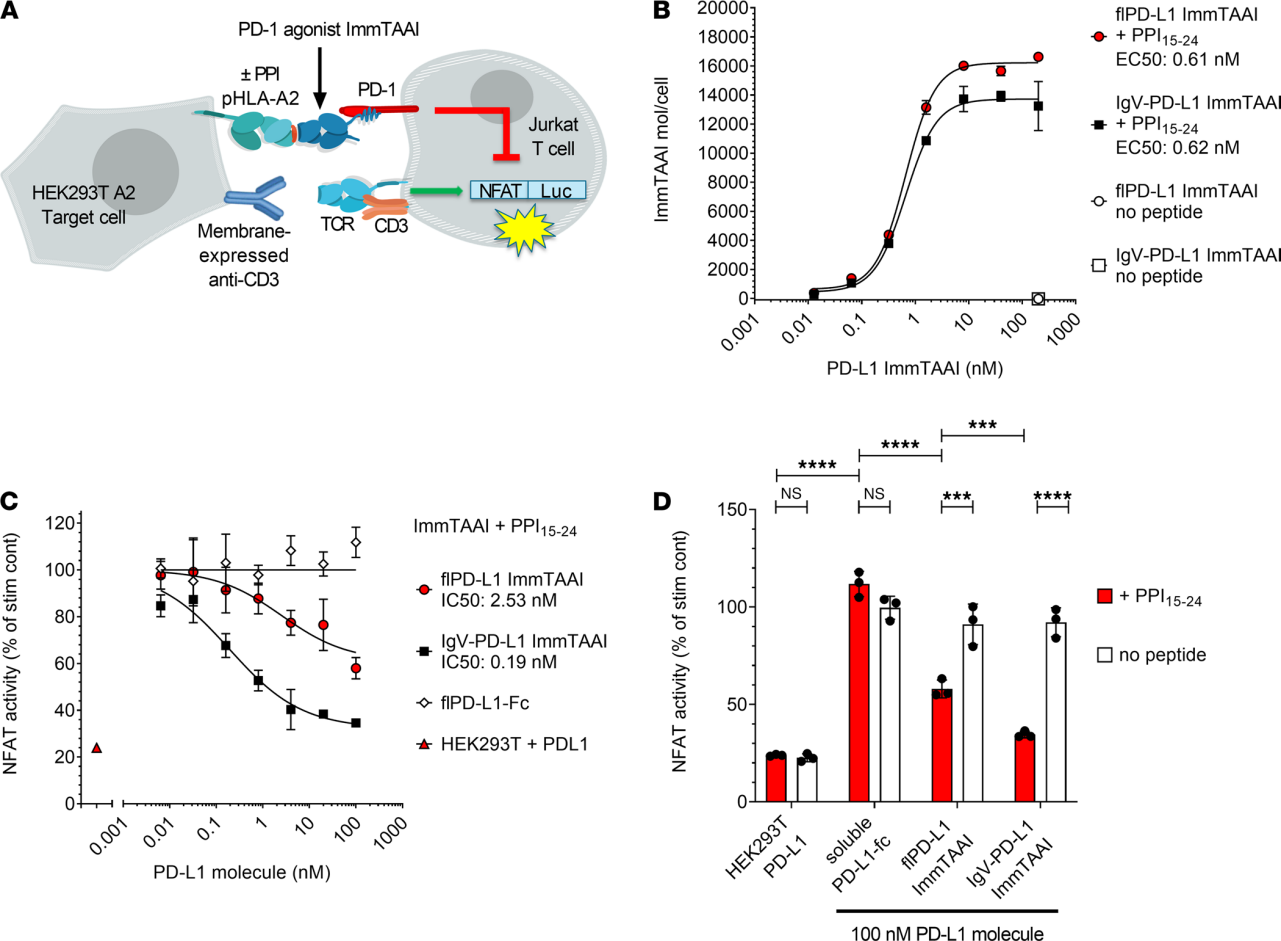
Target cell–bound PD-L1 ImmTAAI molecules inhibit TCR complex signaling. (**A**) Schematic of the HEK293T-A2–Jurkat NFL PD-1 reporter assay. (**B**) PD-L1 ImmTAAI titrations were incubated with PPI_15–24_ peptide–pulsed or nonpulsed HEK293T-A2 target cells. ImmTAAI binding was quantified by flow cytometry, and dose-response curves were plotted (*n =* 3 and representative of 3 independent experiments). (**C**) Dose responses of the PD-L1 ImmTAAIs and PD-L1 Fc were tested in the HEK293T-A2–Jurkat NFL PD-1 reporter assay. Normalized NFAT activity was plotted against ImmTAAI concentration to calculate IC_50_ values (*n =* 3 and representative of 3 independent experiments). (**D**) Relative NFAT activity at 100 nM ImmTAAI was plotted for pulsed (+ PPI_15–24_) versus nonpulsed (no peptide) target cells (*n =* 3 and representative of 3 independent experiments). All data are plotted as mean ± SD and were compared by 2-way ANOVA with repeated measures and Tukey’s or Sidak’s multiple-comparison test. ***P ≤ 0.001, ****P ≤ 0.0001. IgV, immunoglobulin-like variable domain; fl, full-length.

To assess whether target cell binding is necessary for effective PD-1 agonism, PD-L1 containing bispecific ImmTAAI molecules were engineered and tested in a coculture cell assay that allowed direct comparison between target cell–bound versus unbound PD-L1 ImmTAAI. The bispecifics were designed with a targeting end composed of a soluble TCR that is highly specific for a preproinsulin (PPI) peptide, PPI_15–24_, bound to HLA-A*02 on pancreatic β cells (28) and an effector end composed of either a full-length PD-L1 (flPD-L1) or a truncated PD-L1 ligand-binding domain comprising the IgV domain (IgV–PD-L1; Supplemental Figure 1A; supplemental material available online with this article; https://doi.org/10.1172/jci.insight.152468DS1). The cellular coculture assay to measure inhibition of TCR-stimulated T cell signaling was composed of an NFAT-luciferase Jurkat T cell reporter line stably expressing PD-1 (Jurkat NFL PD-1) as effector cell and an engineered HEK293T cell line expressing HLA-A2 β2-microglobulin alone or with PD-L1 as target cell (Figure 1A and Supplemental Figure 1, B and C). HEK293T-A2 target cells were also transiently transfected with membrane-bound anti-CD3 antibody, bypassing the need for endogenous antigen presentation to induce T cell activation (hereafter referred to as HEK293T-A2/anti-CD3). Flow cytometry binding studies demonstrated that the flPD-L1 and IgV–PD-L1 ImmTAAI bispecifics bound to HEK293T-A2/anti-CD3 target cells in a concentration and peptide-dependent manner. In the presence of PPI_15–24_ peptide, maximum binding was achieved below 10 nM and EC_50_ values of 0.61 and 0.62 nM, for flPD-L1 and IgV–PD-L1 ImmTAAI, respectively (Figure 1B).

Coculture of effector and target cells resulted in measurable NFAT activity (Supplemental Figure 1D), while overexpression of PD-L1 on the HEK293T-A2/anti-CD3 target cells resulted in reduction of NFAT activity by more than 75%, highlighting that T cell activation in this assay is sensitive to the PD-1/PD-L1 pathway. In the absence of PD-L1 expression, both flPD-L1 and IgV–PD-L1 ImmTAAI molecules inhibited NFAT activity in a concentration-dependent manner when added to cocultures of effectors and PPI_15–24_ peptide–pulsed target cells (Figure 1, C and D). However, soluble flPD-L1–Fc was completely inactive in this assay, as was PPI_15–24_ TCR alone (Figure 1D and Table 1). The IgV–PD-L1 ImmTAAI was more effective than the flPD-L1 at inhibiting NFAT activity in pulsed cells (inhibition down to 35% versus 58% NFAT activity at 100 nM; *P <* 0.001), with efficacy similar to that observed with PD-L1–expressing HEK293T-A2/anti-CD3 target cells (Supplemental Figure 1D), while neither ImmTAAI molecule inhibited NFAT activity without PPI_15–24_ peptide pulsing of the target cells. Thus, flPD-L1 and IgV–PD-L1 ImmTAAI molecules can inhibit T cells in a target-dependent fashion but only when bound to the target cell, mimicking natural PD-L1–PD-1 engagement.

### PD-1 antibody ImmTAAI molecules are more potent inhibitors of T cell activation than PD-L1 ImmTAAI molecules.

**Figure 2:**
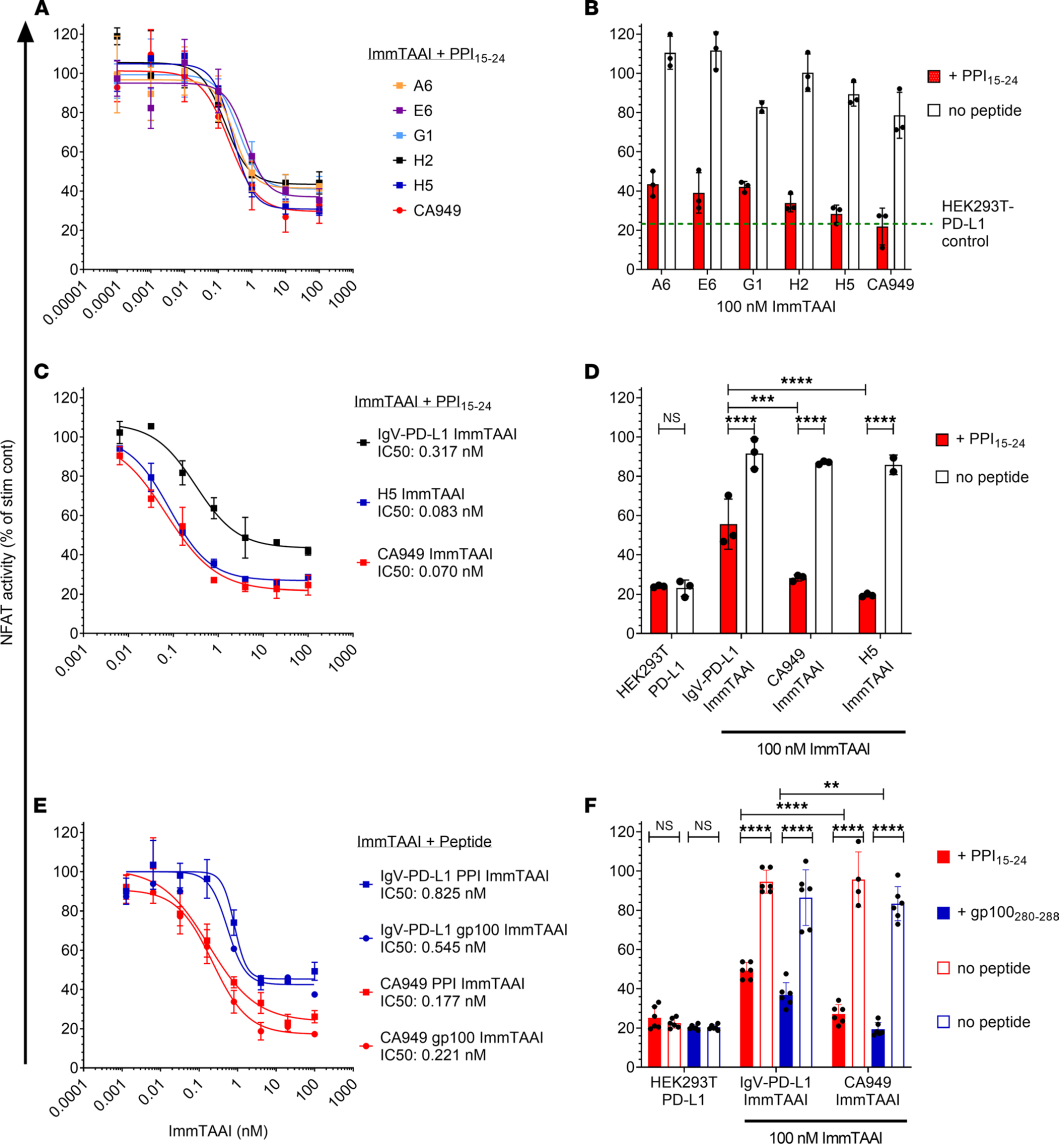
Target cell–bound PD-1 antibody ImmTAAI molecules exhibit superior inhibition of TCR complex signaling over PD-L1 ImmTAAI molecules. (**A **and** B**) PD-1 antibody ImmTAAI molecules constructed using a panel of VHH PD-1 antibodies and an scFv PD-1 antibody (CA949) were tested in the HEK293T-A2/anti-CD3: Jurkat NFL PD-1 reporter assay as described in Figure 1 (*n =* 3 and representative of 3 independent experiments). (**C** and **D**) Representative VHH-based (H5) and scFv-based (CA949) ImmTAAI molecules were tested alongside the IgV–PD-L1 ImmTAAI in the HEK293T-A2/anti-CD3: Jurkat NFL PD-1 reporter assay, and data are plotted as described above (*n =* 3 and representative of 3 independent experiments). (**E **and **F**) PD-1 agonist ImmTAAI molecules were generated by fusing CA949 scFV antibody and IgV–PD-L1 to different TCRs against either gp100_280–288_ pHLA-A2 or PPI_15–24_ pHLA-A2. The ImmTAAI molecules were tested in the HEK293T-A2/anti-CD3: Jurkat NFL PD-1 reporter assay, using HEK293T-A2 target cells pulsed with the appropriate targeting peptide (*n =* 6 and representative of 2 independent experiments). All data are plotted as mean ± SD and were compared by 2-way ANOVA with repeated measures and Tukey’s or Sidak’s multiple-comparison test. **P ≤ 0.01, ***P ≤ 0.001, ****P ≤ 0.0001.

Antibody-based agents possess a number of potential advantages over recombinant PD-L1, including enhanced stability and scope for improved potency, in the generation of clinical therapeutics. As such, we identified several single variable domain on a heavy chain (VHH) PD-1 antibodies with low nanomolar affinities and fused these to the PPI_15–24_ TCR to produce a panel of PD-1 agonist ImmTAAI molecules. The cellular potency of these antibody-based bispecifics was then compared with their PD-L1–containing counterparts in the HEK293T-A2–Jurkat NFL PD-1 reporter assay. Like the PD-L1 ImmTAAI molecules, each of the VHH-based PD-1 agonist ImmTAAI molecules dose dependently inhibited NFAT activity (Figure 2A), measured using the NFAT-luciferase reporter assay described in Figure 1. To explore an alternative antibody format, a single-chain Fv (scFv) fragment (CA949) PD-1 agonist antibody (29) was fused to the PPI_15–24_ TCR. This ImmTAAI caused NFAT inhibition comparable with that observed with VHH-based ImmTAAI molecules. This inhibitory effect was dependent on target cell binding, since no inhibition occurred with soluble VHH or scFv (CA949) antibodies alone or in the absence of peptide (Figure 2B and Table 1).

From the selection of VHH antibodies, H5 was chosen for further studies, as it displayed better potency in some assays. In a direct comparison with the IgV–PD-L1 ImmTAAI, the antibody-based bispecifics H5 and CA949 ImmTAAI exhibited superior potency and efficacy in inhibiting TCR-stimulated NFAT activity in cocultures with peptide-pulsed target cells (Figure 2, C and D, and Table 1). This indicates that bispecific molecules composed of an affinity-enhanced TCR targeting moiety and a potent PD-1 agonist antibody can mimic cellular PD-L1 in promoting PD-1–mediated suppression of T cell activation, providing increased potency compared with the natural ligand.

ImmTAAI molecules are designed to inhibit T cell activation through agonism of the PD-1 pathway — but only when the T cell is in close proximity to the targeted cell type. To ensure that the observed PD-1 agonist activity in a cell-bound state was not unique to the PPI_15–24_ TCR targeting moiety, IgV–PD-L1 and CA949 were each fused to an alternate affinity-enhanced TCR specific for gp100_280–288_ (HLA-A*02:01, YLEPGPVTA), a melanoma epitope (30). Altering TCR specificity had no effect on the PD-1 agonist activity of the ImmTAAI, since each gp100-targeted molecule dose-dependently inhibited TCR-stimulated NFAT activation to a similar level as their PPI-targeted equivalent (Figure 2E), but only when target cells were pulsed with their specific peptide (Figure 2F). These data indicate that, although target cell binding via the TCR targeting domain is essential for ImmTAAI-mediated PD-1 agonism, the incorporated TCR can be varied to achieve specificity for different cell types or tissues, assuming the target epitope is presented in sufficient quantities.

**Figure 3:**
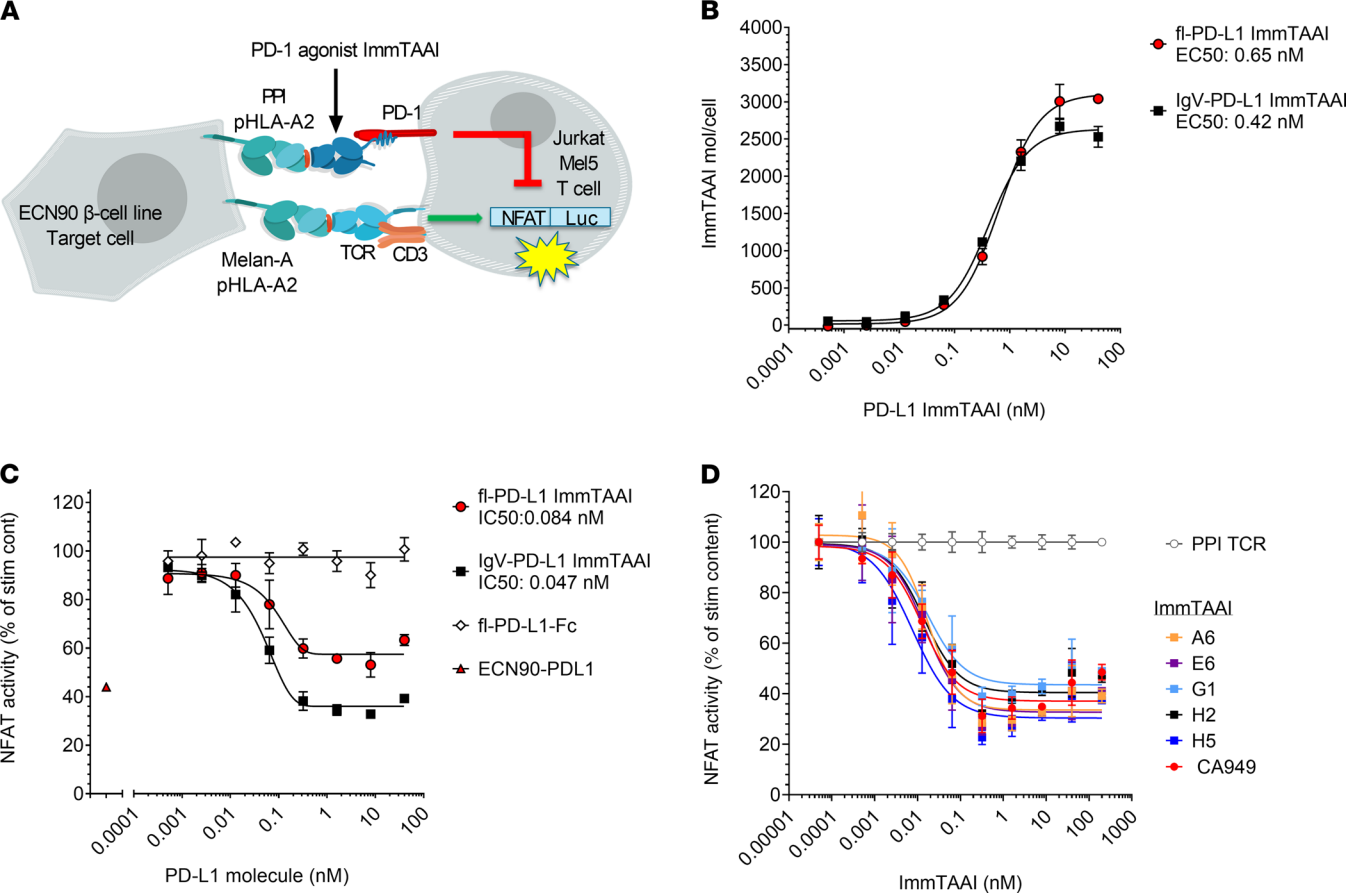
β Cell–targeted PD-1 agonist ImmTAAI molecules inhibit TCR complex signaling. (**A**) Schematic of the ECN90 β cell line: Jurkat NFL Mel5 PD-1 reporter assay. ECN90 cells were pulsed with Melan-A activating peptide, and titrations of PD-L1 or PD-1 Ab ImmTAAI molecules, PD-L1 Fc, or PPI TCR alone added. (**B**–**D**) Either ECN90 cells were incubated for 3 hours and analyzed for PD-L1 ImmTAAI binding (*n =* 3 and representative of 3 independent experiments) (**B**), or Jurkat NFL Mel5 PD-1 cells were immediately added to test activity in the PD-1 reporter assay (*n =* 3 and representative of 3 independent experiments) (**C** and **D**). All data are plotted as mean ± SD. Abbreviations: IgV, Immunoglobulin-like variable domain; fl, full length

To further characterize our ImmTAAI molecules, we designed an alternative NFAT reporter assay incorporating cells with natural peptide HLA (pHLA) target presentation and TCR recognition of cognate antigen. To test whether endogenous levels of PPI_15–24_–HLA-A2 presentation are sufficient to trigger ImmTAAI-mediated PD-1 agonism, the β cell line ECN90, which naturally processes and presents the PPI_15–24_ peptide–HLA-A2 complex, was used as target cells (Figure 3A). Full-length and truncated PD-L1 ImmTAAI molecules specifically bound ECN90 target cells with a similar EC_50_ as that observed for the peptide-pulsed HEK293T-A2 target cells in Figure 1, albeit with a lower total occupancy reflecting the lower natural pHLA levels (Figure 3B).

To provide TCR activation via recognition of cognate antigen, the Jurkat NFL/PD-1 effector cells were transduced with a Mel5 TCR that recognizes the Melan-A_126–135_ (HLA-A*02:01, ELAGIGLTV) epitope (Supplemental Figure 2A). ECN90 target cells were pulsed with Melan-A_126–135_ peptide, enabling physiological T cell activation via natural TCR engagement. NFAT activity downstream of TCR engagement in the TCR transgenic Jurkat effector cell line was efficiently suppressed when cocultured with ECN90 target cells that were induced to overexpress PD-L1 (Supplemental Figure 2, B and C), confirming functionality of this in vitro assay.

In coculture with Melan-A peptide-pulsed ECN90 target cells, the flPD-L1 and IgV–PD-L1 ImmTAAI, as well as the antibody-based PD-1 ImmTAAI molecules, inhibited TCR complex signaling to a similar extent as PD-L1–expressing ECN90 cells (Figure 3, C and D, and Table 2). Consistent with results using peptide-pulsed target cells, the flPD-L1 ImmTAAI showed that the weakest inhibitory activity and soluble forms of each molecule (lacking the TCR targeting domain) failed to inhibit NFAT activity (Figure 3, C and D). Again, antibody-based PD-1 agonist ImmTAAI molecules were more potent than PD-L1–containing bispecifics. Together, these data show that, in a system using physiological T cell activation, target cell–bound antibody-based PD-1 agonists are as effective as natural PD-L1 in inhibiting TCR complex signaling in T cells.

### PD-1 antibody ImmTAAI molecules are noncompetitive with PD-L1 for PD-1 binding and are additive with PD-L1 in suppressing TCR complex signaling.

**Figure 4:**
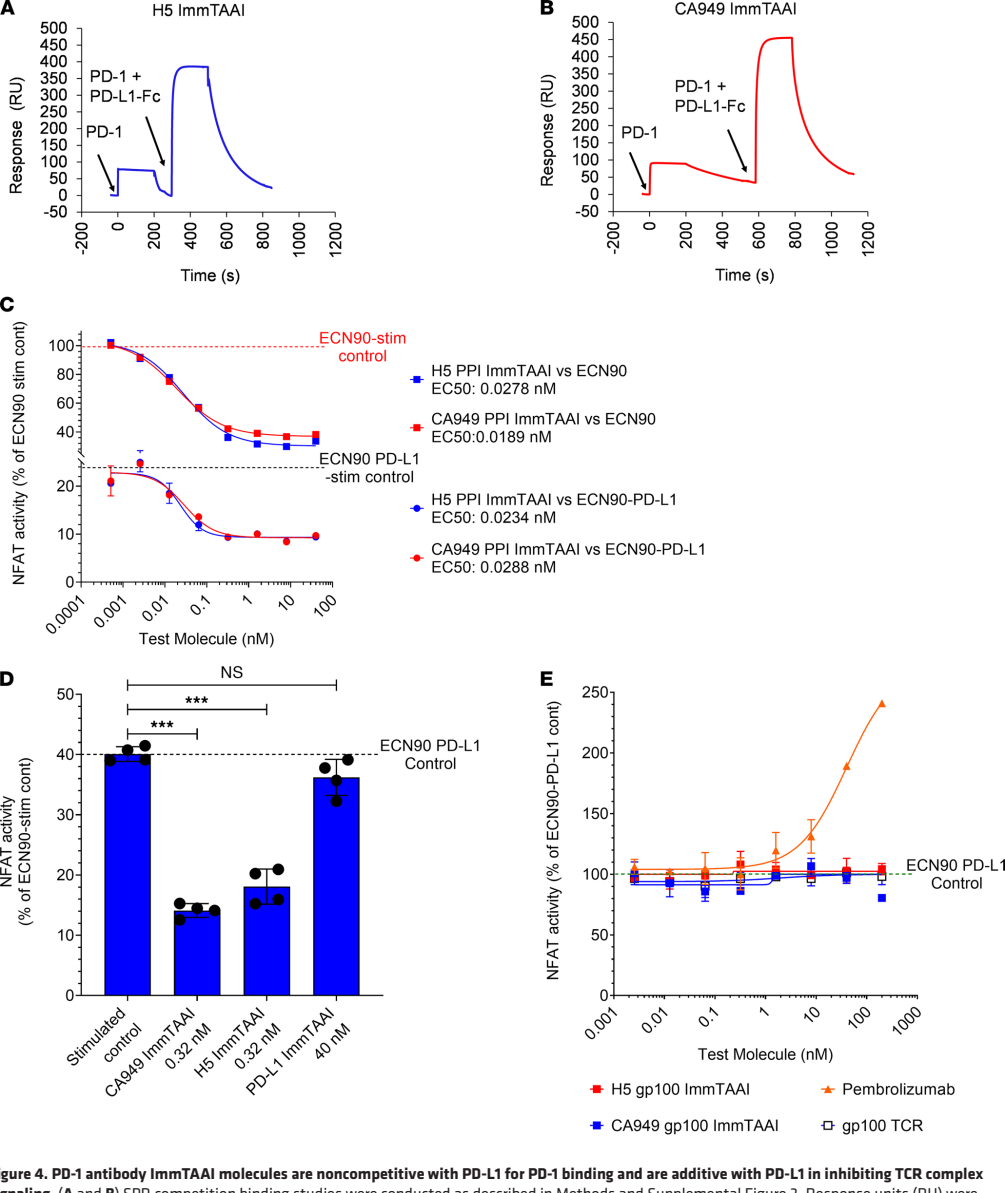
PD-1 antibody ImmTAAI molecules are noncompetitive with PD-L1 for PD-1 binding and are additive with PD-L1 in inhibiting TCR complex signaling. (**A** and **B**) SPR competition binding studies were conducted as described in Methods and Supplemental Figure 3. Response units (RU) were plotted over time to characterize the binding of H5- and CA949-ImmTAAI molecules to PD-1 in the presence of PD-L1–Fc (representative sensorgrams from 3 independent experiments). (**C**) Parental ECN90 (upper curves) or PD-L1 transduced ECN90 cells (lower curves) were pulsed with Melan-A activating peptide and titrations of PD-1 antibody ImmTAAI molecules added. Jurkat NFL Mel5 PD-1 cells were immediately added to run the PD-1 reporter assay (*n =* 2 and representative of 4 independent experiments). (**D**) Cell assay using PD-L1 transduced ECN90 cells was done as described above using titrations of PD-1 antibody ImmTAAI or PD-L1 ImmTAAI molecules. Relative NFAT activity is plotted against the concentrations where maximal inhibition was observed (*n =* 4 and representative of 4 independent experiments). (**E**) Titrations of soluble PD-1 antibody ImmTAAI molecules (with an irrelevant TCR) and a PD-1 blocking antibody (pembrolizumab) were tested in the ECN90–PD-L1: Jurkat NFL Mel5 PD-1 reporter assay (*n =* 2 and representative of 2 independent experiments). Data are plotted as mean ± SD and were compared by 1-way ANOVA and Dunnett’s multiple-comparison test. ***P ≤ 0.001.

Antibodies to PD-1 that do not compete with PD-L1 binding are advantageous for the design of PD-1 agonist T cell inhibitors as they potentially avoid interfering with physiological PD-L1 activity and may even enhance T cell suppression in the presence of PD-L1–expressing target cells. In a competition binding study using surface plasmon resonance (SPR; Supplemental Figure 3), neither the CA949 nor the H5 PD-1 antibody ImmTAAI competed with PD-L1 for PD-1 binding (Figure 4, A and B), presenting the possibility that addition of PD-1 antibody ImmTAAI molecules could enhance PD-L1–mediated T cell inhibition.

To investigate if these bispecifics could enhance PD-L1–driven T cell inhibition, increasing concentrations of either the H5 or CA949-ImmTAAI molecules were added to Jurkat NFL Mel5 PD-1 effector cells cultured with PD-L1–transfected ECN90 target cells. In the absence of ImmTAAI molecules, PD-L1 expression in the ECN90 cells inhibited Mel5 TCR–stimulated NFAT activity by 60%–75% compared with the PD-L1^–^ ECN90 parental line (Figure 4C; top curves). Addition of either the H5 or CA949 PD-1 antibody ImmTAAI molecules to this system dose-dependently enhanced PD-L1–mediated inhibition of NFAT activity, with IC_50_ values of approximately 25 pM. The combined activities of cellular PD-L1 and PD-1 antibody ImmTAAI inhibited NFAT activation by greater than 90% (Figure 4C; bottom curves). Notably, the IC_50_ values of the PD-1 antibody ImmTAAI molecules required here were the same as those observed in the absence of PD-L1 expression (Figure 4C; top curves), indicating that this effect was additive rather than synergistic. In contrast, a PD-L1–containing ImmTAAI had no significant effect on PD-L1–mediated suppression of NFAT activity (Figure 4D). The added inhibition conferred by the PD-1 antibody ImmTAAI is also unlikely due to engagement of additional free PD-1, since the level of PD-L1 expression on the ECN90 β cell line is well in excess of PD-1 expression on the Jurkat T cells (Supplemental Figure 2A and Supplemental Figure 1C).

Conversely, to rule out that the ImmTAAI molecules function as blocking antibodies to PD-L1 in the unbound state, the H5 VHH and CA949 scFv were fused to an irrelevant gp100 TCR that does not recognize peptide-HLA on the surface of ECN90 cells. Both gp100-targeted H5 and CA949 PD-1 antibody ImmTAAI molecules were unable to either interfere with or enhance PD-L1–mediated T cell inhibition in an unbound, soluble state, while pembrolizumab, a well characterized PD-1 antagonist antibody, potently antagonized PD-L1–mediated suppression of TCR-stimulated NFAT activity (Figure 4E). Together, these results highlight the potential for cell-bound PD-1 antibody ImmTAAI molecules to enhance PD-1–mediated suppression of T cell responses in the presence of endogenous PD-L1.

### PD-1 antibody ImmTAAI molecules colocalize with PD-1 at the target cell–effector cell interface and inhibit proximal TCR signaling events.

**Figure 5:**
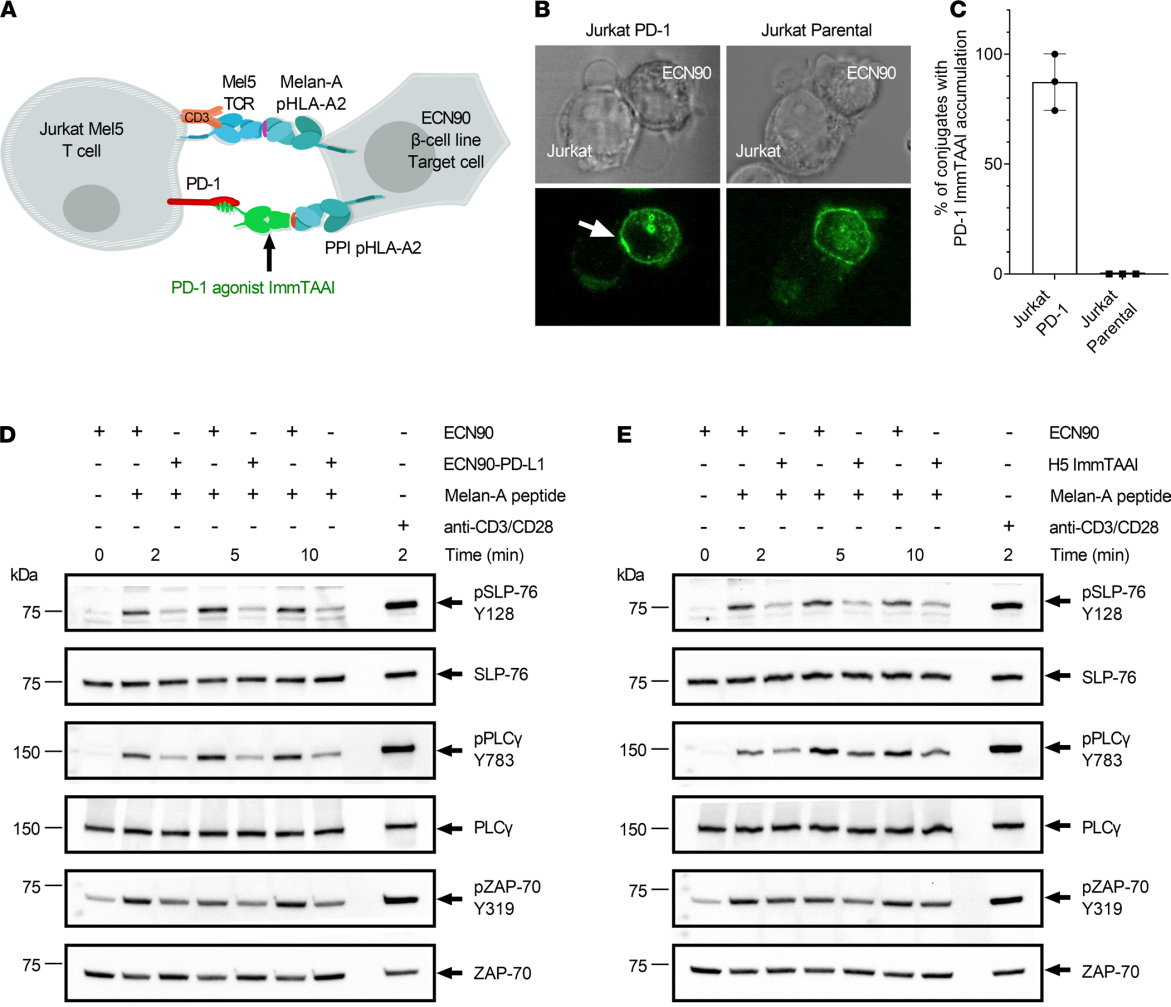
H5 ImmTAAI localizes at the target cell–T cell interface and inhibits proximal TCR signaling. (**A**) Schematic of the cell assay used to visualize H5 ImmTAAI localization. (**B**) Phase contrast and confocal fluorescence images of H5 ImmTAAI localization on ECN90 β cell target cells cocultured with either PD-1^+^ or PD-1^–^ Jurkat cells. Upper panel: phase contrast. Lower panel: fluorescence images, a white arrow indicates ImmTAAI accumulation (representative images from 3 independent studies). Total original magnification, ×100. (**C**) The numbers of ECN90 β cell: Jurkat conjugates with ImmTAAI accumulation (white arrow) were counted and plotted as a percentage of the total number of cell conjugates (*n =* 100 conjugates for conditions using Jurkat Mel5 PD-1 cells and *n =* 96 for Jurkat Mel5 cells, plotted as mean data from 3 independent studies). (**D**) Jurkat Mel5 PD-1 cells were stimulated with Melan-A–pulsed ECN90 or ECN90 PD-L1 cells for the indicated times. Whole cell lysates for each condition and time point were resolved by SDS-PAGE, transferred to PVDF membranes, and probed by Western blotting for phospho–SLP-76 (pSLP-76), total SLP-76 (SLP-76), phospho-PLCγ (pPLCγ), total PLCγ (PLCγ), phospho–ZAP-70 (pZAP-70), and total ZAP-70 (representative blots from 3 independent experiments). (**E**) Jurkat Mel5 PD-1 cells were stimulated with Melan-A–pulsed ECN90 cells, in the presence or absence of H5 ImmTAAI, and Western blots were performed as above (representative blots from 3 independent experiments).

Cellular PD-L1 accumulates at the target cell–T cell interface upon engagement of PD-1 and promotes PD-1/TCR coclustering on the T cell (20, 23). To assess whether the PD-1 agonist ImmTAAI molecules also localize to the T cell–target cell interface, we conducted immunofluorescence studies with labeled ImmTAAI in ECN90/Jurkat Mel5 cocultures. The H5 PD-1 antibody ImmTAAI was fluorescently labeled with Alexa Fluor 488 (AF488), and localization was analyzed by confocal microscopy in cocultures with Melan-A peptide–pulsed ECN90 β cell targets and either PD-1–transduced or nontransduced Jurkat Mel5 effector cells (Figure 5A). The AF488-ImmTAAI bound to the ECN90 target cells and accumulated at the cell-cell interface in cultures with PD-1–expressing Jurkat Mel5 cells in 87% of captured conjugation events (Figure 5, B and C). However, in the absence of PD-1 expression, the ImmTAAI remained dispersed over the surface of the β cell line, and accumulation was not observed for any Jurkat Mel5–target cell conjugates (Figure 5C). This suggests that, as with cellular PD-L1, a targeted synthetic PD-1 agonist accumulates at the T cell–target cell interface upon engagement with PD-1.

We next assessed how this relocalization and accumulation of PD-1 to cell-cell interface upon engagement with target cell–bound ImmTAAI affected proximal TCR signaling. Coculturing the Mel5 TCR transgenic Jurkat T cells with PD-L1–expressing ECN90 target cells resulted in a marked decrease in the phosphorylation of SLP-76, PLCγ, and ZAP-70 in the responding effector T cells, as previously reported (Figure 5D and Supplemental Figure 4, A–C) (23, 25). A similar pattern and extent of inhibition was observed when target cells were preloaded with either the H5 or the CA949 ImmTAAI molecules (Figure 5E and Supplemental Figure 4, D–I), indicating that the PD-1 agonist ImmTAAI molecules are as effective as PD-L1 in inhibiting proximal TCR signaling events. These findings further support the hypothesis that target cell–bound PD-1 antibody ImmTAAI molecules mimic the natural ligand, both in terms of their accumulation at the target cell–T cell interface and their ability to inhibit signaling downstream of TCR engagement.

### PD-1 antibody ImmTAAI molecules inhibit activation of primary human CD4^+^ T cells by antigen-presenting cells.

**Figure 6:**
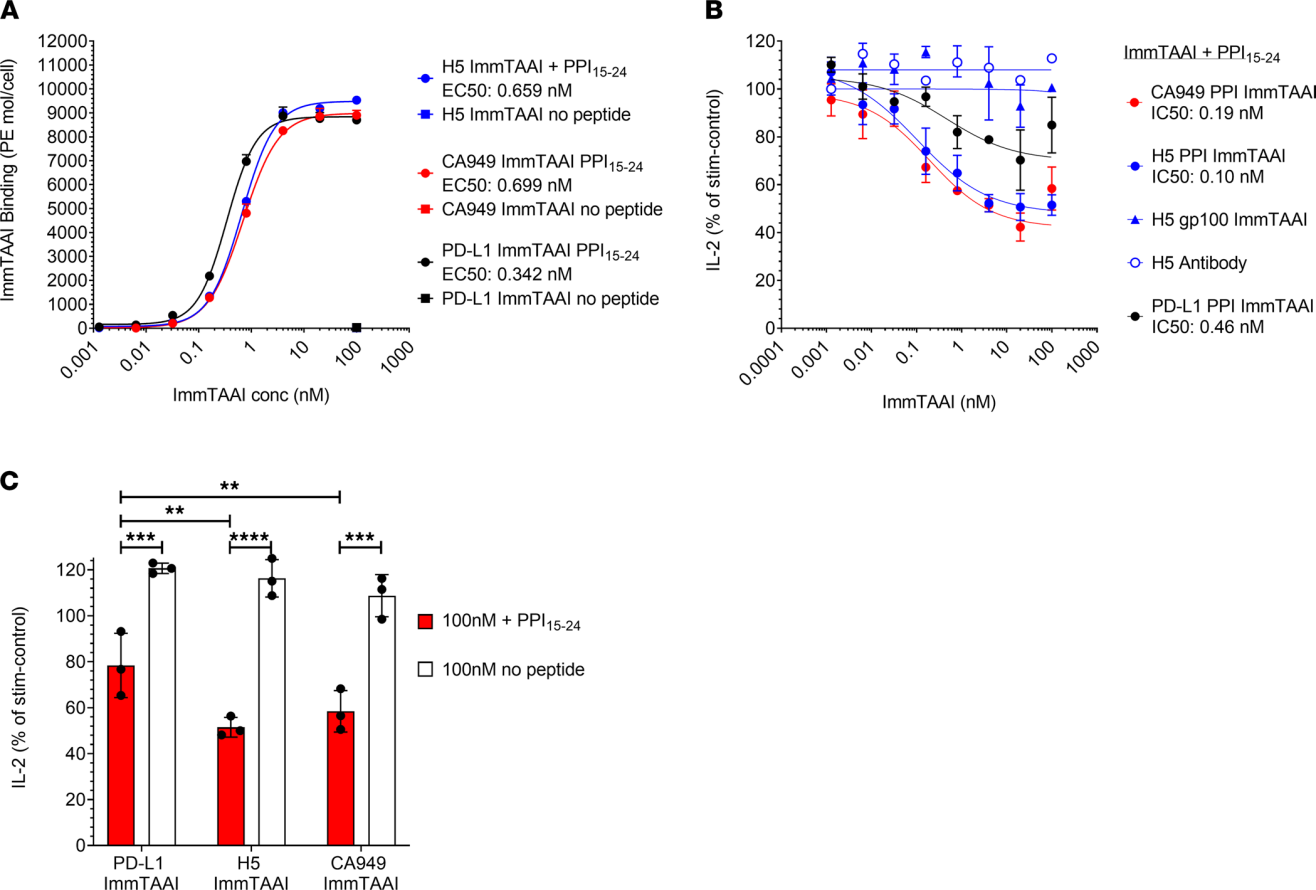
PD-1 antibody ImmTAAI molecules bound to antigen-presenting cells inhibit primary human CD4^+^ T cells. (**A**) PD-1 agonist ImmTAAI binding to Raji-A2 target cells was analyzed by flow cytometry (*n =* 2 and representative of 3 independent experiments). (**B** and **C**) PD-1 agonist ImmTAAI titrations were preincubated for 1 hour with PPI_15–24_ peptide–pulsed or nonpulsed SEB-loaded Raji-A2 cells. Preactivated T cells were added to the Raji-A2 cells and incubated for 48 hours. Supernatants were collected and IL-2 levels measured by ELISA. Dose response curves plotted to obtain IC_50_ values and relative inhibition of IL-2 release at 100 nM ImmTAAI was plotted for pulsed (+ PPI_15–24_) versus nonpulsed target cells (*n =* 3 and representative of 3 independent experiments). Data are plotted as mean ± SD and were compared by 2-way ANOVA with repeated measures and Tukey’s or Sidak’s multiple-comparison test. **P ≤ 0.01, ***P ≤ 0.001, ****P ≤ 0.0001.

The priming of autoreactive CD4^+^ T cells by antigen-presenting cells (APCs) is a key event in many autoimmune diseases. To assess if APC-targeted PD-1 ImmTAAI molecules could provide benefits in such a setting, we generated an assay to determine the impact of these bispecifics on the activation of primary CD4^+^ T cells. Raji cells, which naturally express the CD28 ligands CD80 and CD86 (Supplemental Figure 5A), were used as APCs and transduced with an HLA-A2 β2-microgobulin lentivirus (Raji-A2) to enable PPI_15–24_ peptide presentation and ImmTAAI binding (Figure 6A). For effector cells, primary human T cells, isolated from PBMC, were first stimulated with superantigen-loaded (SEB-loaded) Raji-A2 APCs to induce PD-1 expression (Supplemental Figure 5, B and C). Preactivated CD4^+^PD-1^+^ T cells were subsequently restimulated with unpulsed or PPI_15–24_ peptide–pulsed SEB-loaded Raji-A2 cells to assess PD-1 agonist ImmTAAI activity in either APC-bound or soluble form. In the presence of PPI_15–24_ peptide–pulsed APCs, all PD-1 agonist ImmTAAI molecules reduced IL-2 production by 40%–50% from activated T cells at picomolar levels but were inactive in the presence of nonpulsed APCs (Figure 6, B and C, and Table 3). As in previous assays, the antibody-based ImmTAAI molecules were more potent and efficacious than PD-L1 ImmTAAI molecules, and nontargeted soluble antibodies or antibodies fused to an irrelevant TCR failed to inhibit IL-2 production (Figure 6, B and C). Together, these data build on our findings from reporter-based cell systems and demonstrate that cell-targeted PD-1 agonist ImmTAAI molecules are potent inhibitors of primary CD4^+^ T cell activation.

### PD-1 antibody ImmTAAI molecules inhibit autoreactive human CD8^+^ T cell activation and protect target cells from T cell killing.

**Figure 7:**
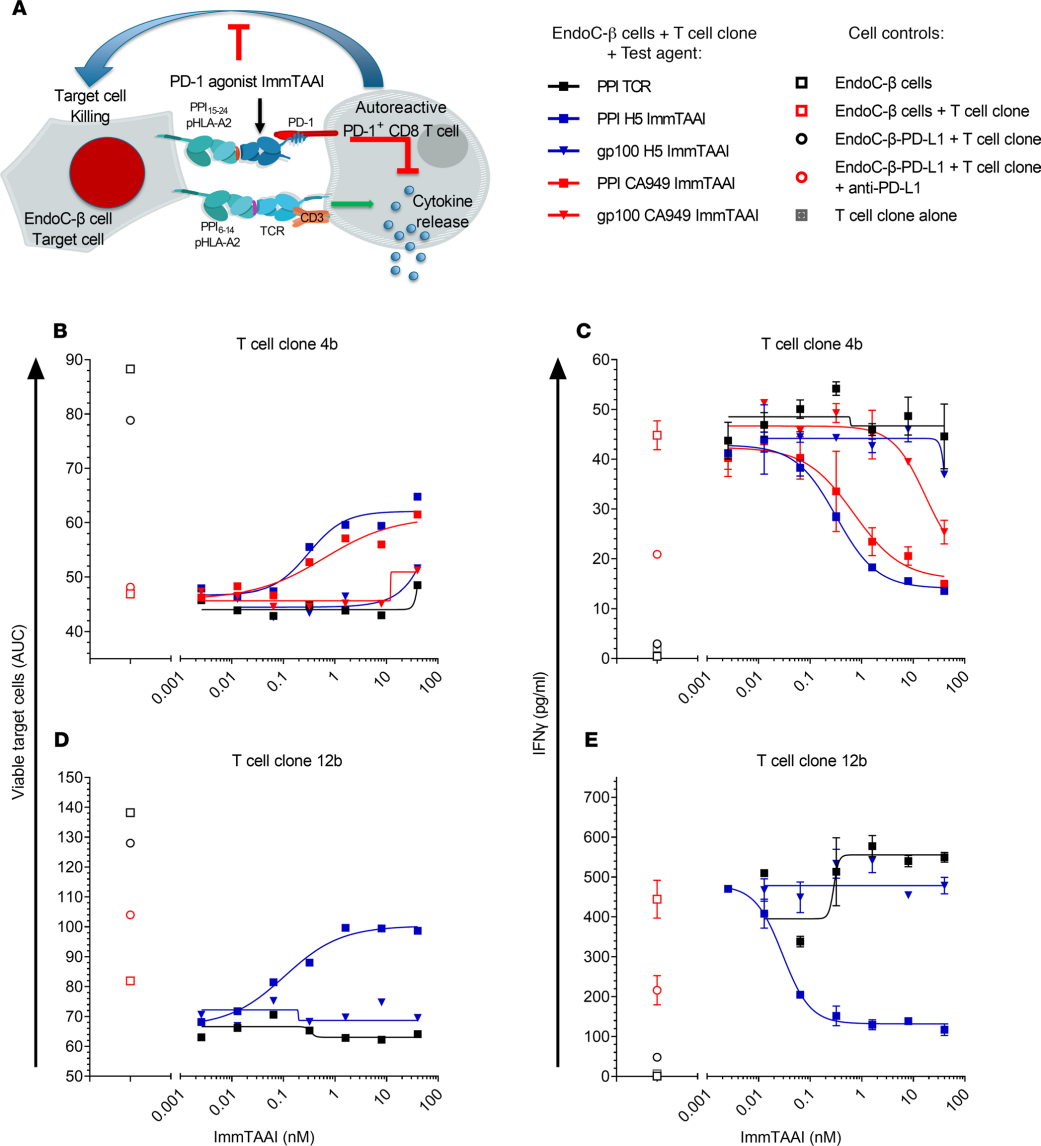
PD-1 antibody ImmTAAI molecules inhibit autoreactive human CD8^+^ T cells and protect target cells from T cell killing. (**A**) Schematic of the EndoC-β Red cell–CD8^+^ T cell clone killing assay. (**B** and **D**) PPI_6–14_-HLA-A2-specific autoreactive T cell clones 4b (**B**) or 12b (**D**) were added to EndoC-β Red cells in the presence of PPI antibody or gp100 PD-1 antibody ImmTAAI titrations or TCR only controls. PD-L1–transduced EndoC-β Red target cells with or without anti–PD-L1 blocking antibody were used as additional controls. For each sample EndoC-β Red target cell number, relative to cells at (t = 0) was measured over time by imaging and growth curves generated. AUC was calculated for each curve and EC_50_ data generated by plotting AUC over ImmTAAI concentration (representative data from 3 independent experiments). (**C** and **E**) Supernatants were collected from the killing assays at 24 hours. IFN-γ levels were measured by MSD ELISA, and dose-response curves were plotted to generate EC_50_ values. Nonstimulated T cells alone were assessed as additional controls (*n =* 2, data are plotted as mean ± SD and representative of 3 independent experiments).

To explore if PD-1 agonist ImmTAAI molecules can inhibit autoreactive CD8^+^ T cells, a T cell cytotoxicity assay was developed using the pancreatic β cell line EndoC-βH2-A2, which naturally presents the PPI_15–24_–pHLA epitope recognized by the PD-1 agonist ImmTAAI molecules, as target cells and using CD8^+^ T cell clones that recognize a distinct pancreatic β cell antigen PPI_6–14_ (RLLPLLALL)–HLA-A2 as effectors (Figure 7A). Cell viability was assessed over time by Incucyte imaging and by labeling target cells with NucLight red to generate EndoC-βH2 Red target cells. Initial cytotoxicity experiments, conducted using a T cell clone with low TCR affinity to PPI_6–14_ pHLA-A2 (clone 4b; *K_D_* ~800 μM; Table 4), show that clone 4b reduced target β cell viability by approximately 50% at an effector to target (E:T) ratio of 1:1. This decrease in cell viability was almost completely reversed when T cells were cultured with PD-L1–overexpressing target EndoC-βH2 Red cells (Figure 7, B and C). Likewise, addition of either the H5 or CA949 ImmTAAI molecules also reduced T cell–mediated cytotoxicity with picomolar potency (Figure 7B; Supplemental Figure 6, A and B; Supplemental Videos 1 and 2; and Table 5). This effect was dependent on specific ImmTAAI targeting to the EndoC-βH2 Red target cells, as PD-1 antibody ImmTAAI molecules containing an irrelevant gp100 TCR showed minimal activity. The PPI_15–24_ TCR alone also had no effect, providing further evidence that the protective activity of the PPI-targeted ImmTAAI molecules was due to PD-1 agonist activity.

Similar results were obtained in a separate experiment using a more potent T cell clone with a higher-affinity TCR to PPI_6–14_ pHLA-A2 (clone 12b, *K_D_* 90 μM; Table 4). Clone 12b T cells required 100-fold less target PPI_6–14_ peptide and a lower E:T ratio than clone 4b to achieve a similar level of target cell killing (Figure 7D). Again, the H5 ImmTAAI dose-dependently protected EndoC-βH2 Red cells from clone 12b T cell–mediated killing with picomolar potency (Figure 7D; Supplemental Figure 6D), increasing cell viability by approximately 50% above the level observed with maximum cell killing.

Cell culture supernatants from both coculture assays were also assessed for cytokine production. In addition to protecting the β cell line from CD8^+^ T cell clone killing, target cell–bound bispecifics potently inhibited IFN-γ (Figure 7, C and E, and Table 5) and TNF-α production (Supplemental Figure 6C). These data demonstrate that cell-bound PD-1 agonist ImmTAAI molecules potently inhibit CD8^+^ T cell cytokine secretion and confer protection from autoreactive CD8^+^ T cell–mediated cytotoxicity in vitro.

## Discussion

PD-1 agonist ImmTAAI molecules are TCR bispecifics designed to inhibit T cell activation, through agonism of the PD-1/PD-L1 pathway but only when the T cell is in a specific organ or tissue. To maximize their effectiveness, they have been engineered to mimic cellular PD-L1 and engage PD-1 at the target cell–T cell interface. Here, we show that cell surface presentation of these PD-1 agonists (via their TCR domain), whether based on PD-L1 itself or potent agonistic antibodies, is essential to their activity, as only target cell–bound ImmTAAI molecules were able to inhibit T cell function. Therefore, this study outlines a design strategy to create efficacious synthetic PD-1 agonist molecules that only confer local immune suppression in the tissue under attack, providing an advantage over systemically acting immune modulators.

This study provides proof of principle that target cell presentation is an important component of the ability of PD-L1 to effectively activate the PD-1 pathway (20, 24, 25, 31). The observation that an ImmTAAI constructed with IgV–PD-L1 was more potent and effective than a flPD-L1 ImmTAAI suggests that the size and orientation of cell-targeted PD-1 agonists is also important for optimal PD-1 activity (32–34). These data are consistent with a previous report, in which lengthening the extracellular domain of PD-1 with additional Ig domains led to incremental reductions in both PD-1 colocalization to TCR microclusters and inhibition of IL-2 production (23).

Antibody-based ImmTAAI molecules were also able to recapitulate the mechanism of PD-L1–mediated PD-1 activation by localizing at the target cell–T cell interface and inhibiting proximal TCR signaling events. However, they were more potent and efficacious than their PD-L1–based counterparts in both reporter and functional assays. Although the mechanisms responsible for this are not fully understood, it is possible that the nanomolar binding affinity of the PD-1 antibodies used in our ImmTAAI molecules influences the strength of PD-1 agonism. The natural affinity of PD-L1 for PD-1 is in the low micromolar range (35, 36), and effective PD-1–mediated suppression of T cell responses appears to require high levels of PD-1 and PD-L1 expression together with high densities of PD-1–PD-L1 complexes at the T cell–target cell interface (31, 37). Such high-order interactions are likely to be necessary to promote PD-1 signaling by providing stability to the PD-1–PD-L1 complexes through increased avidity. The strong affinity of the PD-1 antibodies used in our studies could lead to more stable PD-1 antibody–PD-1 interactions and initiate signaling at a lower receptor occupancy.

Antibody-based ImmTAAI molecules were not only more potent, but also provide additional advantages over PD-L1–based agonists. First, they do not affect the binding of cellular PD-L1 to PD-1 on an interacting T cell. This feature is not only crucial with regard to lack of interference with physiological tolerance signaling — e.g., in the intestine or placenta — but is of particular relevance in the context of autoimmunity and chronic inflammation, as PD-L1 expression is stimulated by inflammatory cytokines and upregulation of PD-L1 has been observed in certain autoimmune diseases, such as type 1 diabetes (38). Antibody-based ImmTAAI molecules were also additive with cellular PD-L1 in triggering PD-1 activity on T cells, suggesting that our targeted approach could further promote endogenous defence mechanisms during inflammation. Furthermore, the antibody-based ImmTAAI molecules do not act as PD-L1–PD-1 antagonists in solution, something that has been suggested for soluble PD-L1 in autoimmune disease (39, 40).

Recent studies have shown that there are additional levels of complexity in the regulation of PD-L1–PD-1 signaling in T cells. PD-L1 is able to bind to CD80 in *cis* on APCs, and this interaction prevents PD-L1–PD-1 binding (20, 41–43). PD-L1 can also bind to PD-1 in *cis* on both APCs and T cells, which either neutralizes or promotes PD-1 signaling in T cells, respectively (44, 45). While all of these mechanisms may influence the ability of a PD-L1–based bispecific to promote PD-1 signaling in autoimmune settings, PD-1 antibody–based ImmTAAI molecules can operate as tissue-targeted PD-1 agonists irrespective of CD80 or PD-L1 status.

There is currently some controversy regarding the relative roles of the TCR and CD28 signaling pathways in PD-1–mediated inhibition of T cell function, with some studies reporting a requirement for CD28 for effective PD-1–mediated T cell inhibition and others indicating that CD28 signaling reduces the ability of PD-1 to suppress T cell function (24, 46–48). Despite these disparities, cell-targeted PD-1 antibody ImmTAAI molecules inhibit primary human T cells, irrespective of the presence or absence of CD28 costimulation. While our research has focused on the ability to trigger the PD-1 pathway in CD4^+^ and CD8^+^ effector T cells, it is important to note that PD-1 is expressed on various T cell subsets and other immune cells, including NK cells, B cells ,and macrophages, so it will be important to assess the scope of PD-1 agonists to regulate these different cell types (49–56).

Studies using primary human T cells allowed the investigation of PD-1 agonist ImmTAAI activity in more physiological settings. Since both the PD-1 agonist and the TCR targeting domain of our molecules are human specific, we did not attempt to use a murine in vivo model (e.g., NOD mice); instead, we generated several in vitro systems to study the effect of PD-1 agonist ImmTAAI molecules on human primary CD4^+^ and CD8^+^ T cell function. The Raji-A2 SEB T cell assay models the activation of an antigen-experienced T cell population, predominantly CD4^+^PD-1^+^ T cells, by APCs while the killing studies with insulin-specific CD8^+^ T cell clones model the recognition of self-peptide HLA on pancreatic islet β cells by autoreactive CD8^+^ T cells and ensuing β cell destruction that occurs in type 1 diabetes. In both models, ImmTAAI molecules were able to potently and efficaciously inhibit T cell function.

While our studies provide proof of principle for the inhibitory activity of ImmTAAI molecules, further experiments are warranted to understand the scope of these bispecifics more fully. For example, models of APC-mediated activation of autoreactive or allergen-specific CD4^+^ T cells may more closely reflect the pathological processes in diseases where these cells predominate. Similarly, CD4^+^ and CD8^+^ T cell models incorporating several different T cell clones with distinct specificities could be used to test the ability of PD-1 agonist ImmTAAI molecules to simultaneously suppress multiple T cell reactivities against different antigens on APCs or target cells. Another important question is whether ImmTAAI molecules induce any long-lasting effects on the phenotype and function of T cells, as such prolonged changes could impact significantly on pharmacodynamics and efficacy. We therefore plan to investigate the long-term effects of our bispecifics on T cell function by looking at markers of anergy, exhaustion, and death.

In summary, we have shown that binding a PD-1 agonist to the surface of target cells is a highly effective way to activate the PD-1 receptor on interacting T cells and achieve immune suppression. Potent PD-1 antibody ImmTAAI molecules closely mimic the mechanism of action of endogenously expressed PD-L1 in their localization to the target cell–T cell interface, inhibition of proximal TCR signaling events, and ultimately suppression of T cell function. Crucially, in soluble form, the PD-1 ImmTAAI molecules are inactive; they neither activate PD-1 on T cells nor block PD-L1–PD-1 signaling, thus avoiding both systemic immunosuppression and the risk of immune-related adverse events. Our studies highlight a path forward to generate more effective, potent PD-1 agonists that can inhibit T cell function, are biologically inactive in solution, and have scope to treat autoimmune diseases, an area of high unmet need, where effective and safe new therapies are urgently needed.

## Methods

### Human PBMC and cell lines.

PBMC used in this study were obtained from healthy volunteers (Immunocore employees). The Jurkat NFAT luciferase T cell line was purchased from Promega and maintained in RPMI-1640 supplemented with 10% heat-inactivated FBS, 2 mM L-glutamine, 0.1 mM MEM nonessential amino acids, 1 mM sodium pyruvate, pen/strep (50 U/mL penicillin and 50 µg/mL strep), and 200 μg/mL hygromycin B. The Raji B cell lymphoma line was purchased from ATCC and maintained in R10 growth media (RPMI-1640 supplemented with 10% heat-inactivated FBS, 2 mM L-glutamine, and pen/strep). HEK-293T cells were purchased from ATCC and maintained in DMEM (4.5 g/L glucose) supplemented with 10% heat-inactivated FBS and 2 mM L-glutamine. The immortalized human pancreatic β cell lines, ECN90 and EndoC-βH2, were purchased from Univercell Biosolutions and maintained in Optiβ3 and Optiβ1 media, respectively, in tissue culture vessels precoated with β-coat (Univercell Biosolutions).

All standard media (RPMI-1640, DMEM), FBS, glutamine, MEM nonessential amino acids, sodium pyruvate, pen/strep, PHA, and PBS were purchased from Thermo Fisher Scientific. Hygromycin B was purchased from Invitrogen. Dithiothreitol was from Sigma-Aldrich.

### TCR engineering.

TCRs targeting PPI_15–24_ (HLA-A*02:01, ALWGPDPAAA) or gp100_280–288_ (HLA-A*02:01, YLEPGPVTA) peptide HLAs were isolated either from in-house phage libraries using standard phage-display methods (57) or from T cell cloning using human donors. TCRs were then affinity enhanced through molecular evolution using phage display as previously described (58).

### Agonist PD-1 antibody generation.

PD-1–specific antibodies were generated by immunization of llamas with recombinant human PD-1–His protein (Acro Biosystems, PD1-H5221) and after 2–3 rounds of phage-display panning on immobilized recombinant human PD-1–Fc protein (Acro Biosystems, PD1- H5257), using adapted methods from literature (59).

### ImmTAAI production.

ImmTAAI molecules were generated by fusing the affinity-matured TCRs to the desired PD-1 agonist effector moiety. Effector moieties included truncated PD-L1 comprising the IgV domain (residues 19–134), PD-L1 full extracellular domain, an scFv PD-1 agonist antibody (CA949), and VHH PD-1 agonist antibodies. The resulting constructs were produced either by expression as inclusion bodies in *E. coli* followed by refolding and purification as described previously (60), or soluble expression in mammalian cells (Expi-CHO-S, Thermo Fisher Scientific) followed by purification using immobilized metal affinity chromatography and size exclusion chromatography.

### Lentivirus production and transduction.

Lentiviral vectors containing *PDCD1* (PD-1) and *CD274* (PD-L1) were obtained from OriGene (RC210364L1 and RC213071L1, respectively). NucLight red (IncuCyte NucLight Red Lentivirus Reagent) was obtained from Essen Bioscience. Lentiviral vectors for HLA-A2 β2-microglobulin and the Mel5 TCR were produced in-house. Lentiviruses were packaged by transfection of lentiviral vectors with packaging plasmids (pMD2.g, pMDLg/p RRE, and pRSV.REV, in-house) into HEK293T cells with TurboFect Transfection Reagent (Thermo Fisher Scientific). Lentiviral particles were collected 48 hours after transfection, filtered through a 0.45 μm filter (Nalgene), and concentrated by centrifugation at 10,000*g* for 16 hours at 4°C. Lentivirus pellets were resuspended in growth media and stored at –80°C prior to use. For transduction of cell lines, lentivirus was added to 1 × 10^6^ of exponentially growing cells in wells of a 24-well cell culture plate. After 48–72 hours, transduced cells were harvested, assessed for expression of transduced genes by flow cytometry, and expanded into cell culture flasks. Where necessary, cells were sorted by FACS or enriched using anti-PE beads (Miltenyi Biotec), to obtain populations of cells > 95% positive for the transduced gene product of interest. For transduction of T cell clones with PD-1 lentivirus, 1 × 10^6^ T cell clones were stimulated with a mix of irradiated PBMC from 4 donors in the presence of 2 μg/mL PHA and 100 U/mL recombinant human IL-2 (Peprotech) for 24 hours. Lentivirus was added to the coculture and incubated for several days. Transduction efficiency was evaluated by flow cytometry. PD-1^+^ T cell clones were sorted with (Sony Biotechnology), and cells were reexpanded with feeders in the presence of PHA and recombinant IL-2 at 100 U/mL. T cell clones were used in the killing assay after 20 days.

### Jurkat NFAT luciferase PD-1 reporter assays.

HEK293T: Jurkat NFL PD-1 reporter assay, HEK293T-A2 target cells, were washed and resuspended in D10 media without antibiotics (DMEM, 10% FBS) and plated into white 96-well cell culture plates (50,000 cells/well) at 37°C, 5% CO_2_ for 24 hours. The HEK293T cells were transfected with 100 ng membrane-bound anti-CD3 antibody plasmid or with 100 ng membrane-bound anti-CD3 antibody + PD-L1 plasmid (BPS Bioscience) and 0.3 μL Lipofectamine 2000 (Invitrogen) per well, using Opti-MEM I (Thermo Fisher Scientific) as diluent. After 24 hours, the cells were either pulsed by adding D10 media containing 20 μM PPI peptide (ALWGPDPAAA) or mock-treated with D10 media alone. After incubating for 2 hours at 37°C, 5% CO_2_, media was removed from each well, and assay buffer alone (RPMI-1640, supplemented with 10% heat-inactivated FBS and 2 mM L-glutamine) or assay buffer containing titrations of PD-1 agonist ImmTAAI molecules or PD-L1 Fc (R&D Systems) was added to each well. The assay was initiated by adding 50,000 Jurkat NFL PD-1 effector cells in assay buffer and incubating for 16–20 hours at 37°C, 5% CO_2_.

ECN90–Jurkat NFL Mel5 PD-1 reporter assay — ECN90 or ECN90–PD-L1 cells were harvested and plated at 50,000 cells/well in Optiβ3 media, into the inner 60 wells of a white 96-well cell culture plate that had been precoated with β-coat (Univercell Biosolutions). After incubating at 37°C, 5% CO_2_ for 16–20 hours, media was removed and assay buffer containing 10 μM Melan-A peptide (ELAGIGILTV) was added. After pulsing for 2 hours at 37°C, 5% CO_2_, assay buffer alone or assay buffer containing titrations of PD-1 agonist ImmTAAI molecules was added to each well. The assay was initiated by immediately adding 50,000 Jurkat NFL Mel5 PD-1 effector cells and incubating for 16–20 hours at 37°C, 5% CO_2_.

Both reporter assays were developed by adding BioGlo reagent (Promega), and NFAT activity was determined by measuring luciferase luminescence on a plate reader (Clariostar, BMG Labtech). NFAT activity was normalized against TCR-stimulated controls and ImmTAAI dose-response data were analyzed in Prism (GraphPad) using a 4 parameter, nonlinear least squares fit to determine IC_50_ values. For the HEK293T: Jurkat NFL PD-1 reporter assays, relative NFAT activity at 100 nM ImmTAAI for pulsed (+PPI_15–24_) versus nonpulsed (no peptide) target cells was also plotted to highlight the targeting-dependency of PD-1 agonist ImmTAAI-mediated inhibition.

### Primary human T cell IL-2 assay.

Primary human T cells were isolated from PBMCs using a pan T cell isolation kit (Miltenyi Biotec) and resuspended in R10. The T cells were preactivated by incubating with irradiated Raji A2 cells, preloaded with 1 µg/mL SEB (MilliporeSigma), using 1 × 10^6^ T cells to 1 × 10^6^ Raji cells per well of 24-well tissue culture plates. Preactivation plates were incubated for 10 days at 37°C, 5% CO_2_ and IL-2 (50 U/mL) added at days 3 and 7. After preactivation, the expanded T cells were predominantly CD4^+^ T cells and typically 60%–70% PD-1^+^. The T cells were washed 3 times in R10 to remove IL-2 and dead cell debris, and they were resuspended in fresh R10. For restimulation of preactivated T cells in the presence of target cell–bound or soluble PD-1 agonist ImmTAAI molecules, Raji-A2 cells were pulsed, or not, with 20 μM PPI_15–24_ peptide at 2 × 10^6^ cell/mL in R10 for 2 hours at 37°C, 5% CO_2_. Raji-A2 and Raji-A2–PD-L1 cells were then loaded with 31.6 ng/mL SEB for 1 hour at 37°C, 5% CO_2_, and irradiated with 33 Gy. Raji-A2 and Raji-A2–PD-L1 cells were plated into the inner 60 wells of flat-bottomed 96-well culture plates at 100,000 cells/well. R10 alone or R10 containing PD-1 agonist ImmTAAI dilutions was added to PPI pulsed or nonpulsed Raji-A2 cells. For PD-L1 inhibition controls, R10 alone or R10 containing a PD-L1 blocking antibody at a final concentration of 12.5 μg/mL (BioLegend, catalog 329716, clone 29E.2A3) was added to Raji-A2–PD-L1 cells. After 1 hour preincubation, washed preactivated T cells were added to the Raji-A2 cells at 100,000 cells/well and incubated for 48 hours at 37°C, 5% CO_2_. Supernatants were collected, and IL-2 levels were measured by ELISA (IL2 Ready-SET-Go! ELISA, Invitrogen). IL-2 release was normalized against SEB-stimulated controls, and ImmTAAI dose response data were analyzed in Prism (GraphPad) using a 4 parameter, nonlinear least squares fit to determine IC_50_ values. Relative IL-2 levels at 100 nM ImmTAAI for pulsed (+ PPI_15–24_) versus nonpulsed (no peptide) target cells were also plotted to highlight the targeting-dependency of PD-1 agonist ImmTAAI-mediated inhibition.

### ImmTAAI colocalization studies.

H5 PPI ImmTAAI was labeled with AF488 using the CF488A SE Protein Labeling Kit (Biotium) according to the manufacturer’s instructions. ECN90 cells were harvested, washed in PBS 10% FBS, and resuspended at 1 × 10^6^ cells/mL in OpitiB3 (Univercell Biosolutions). Cells were pulsed with 2.5 μM Melan-A peptide for 2 hours, washed with PBS, and resuspended at 1 × 10^6^ cells/mL in PBS 2% FBS. ECN90 cells were incubated with 100nM AF488-ImmTAAI at room temperature for 30 minutes. Excess ImmTAAI was washed away with PBS 2% FBS and resuspended in RPMI (no FBS) at 0.8 × 10^6^ cells/mL in RPMI. ECN90 cells were added to glass slide chambers (Ibidi) at 80,000 cells/well, incubated at 37°C for 10 minutes. Jurkat NFL Mel5 parental and PD-1 cells were resuspended at 0.8 × 10^6^ cells/mL in R10 and added to ECN90 cells at a 1:1 ratio (80,000 Jurkat cells/sample) and incubated for 30 minutes at 37°C. Cells were fixed for 20 minutes with Fixative BD stabilizing. The reaction was quenched by washing with PBS 2% FBS. *Z* stack images were acquired using a confocal microscope, Nikon (Nikon Eclipse Ti2 and Nikon A1 confocal system), and Imaging analysis was performed with Image J (Fiji). For each condition (Jurkat NFL Mel5 PD-1 or Jurkat NFL Mel5 parental cells), the number of ECN90–Jurkat conjugates exhibiting accumulation of AF488-ImmTAAI was counted and plotted as a percentage of the total number of conjugates.

### TCR stimulation and Western blot studies.

ECN90 and ECN90–PD-L1 cells were harvested by washing twice in PBS and resuspending in nonenzymatic cell dissociation buffer (Thermo Fisher Scientific). ECN90 and ECN90–PD-L1 cells were resuspended at 2 × 10^6^/mL in Optiβ3 and pulsed, or not, with 10 μM Melan-A peptide for 2 hours at 37°C on a rotator. After pulsing, cell suspensions were diluted 1:2 with either media alone or media containing 50 nM PD-1 antibody ImmTAAI. ECN90 cells with or without PD-1 antibody ImmTAAI were incubated on a rotator for 2 hours at 37°C. Unbound PD-1 antibody ImmTAAI was removed by washing the ECN90 cells twice in RPMI-1640 and resuspended at 20 × 10^6^/mL in RPMI alone by removing the required volume of media. During the preparation of the ECN90 target cells, Jurkat NFL Mel5 PD-1 effector cells were washed twice in RPMI-1640, resuspended in RPMI alone at 20 × 10^6^ cells/mL, and starved at 37°C for 4 hours on a rotator. Stimulation time course studies with Melan-A-pulsed target cells and Jurkat Mel5 cells established that maximal TCR-stimulated phosphorylation of the signaling molecules SLP-76, PLCγ, and ZAP-70 occurred within a 10-minute time frame. TCR stimulations were initiated by adding 250 μL Jurkat NFL Mel5 PD-1 cells at 20 × 10^6^/mL to 250 μL ECN90 cells in 1.5 mL Eppendorf tubes and pelleting at 400*g* for 10 seconds to promote effector cell/target cell contact. Stimulations were conducted over a 10-minute time-course and stopped by spinning down at 17,000*g* in a microfuge for 12 seconds, removing the supernatant and adding 250 μL NP-40 lysis buffer (Alfa Aesar), supplemented with protease inhibitor cocktail (MilliporeSigma) and phosphatase inhibitor cocktail (PhosSTOP, Roche). Cells were lysed on ice for 20 minutes, and nonsoluble material was pelleted by spinning at 20,000*g* in a microfuge at 4°C for 10 minutes. Protein levels of clarified cell lysates was determined by BCA assay (Thermo Fisher Scientific), and whole cell lysates were prepared by adding the clarified lysates to 4× Laemmli sample buffer (Bio-Rad) containing 200 mM dithiothreitol. Samples were denatured at 95°C for 5 minutes and then stored at –80°C for subsequent Western blotting. Samples were run on denaturing 4%–20% SDS-PAGE gels (Bio-Rad) at 50 μg protein/sample and transferred to PVDF membranes (Bio-Rad). Membranes were probed with primary antibodies specific for phospho–SLP-76 (pY128, BD Pharmingen, catalog 558367, clone J141-668.36.58), phospho-PLCγ (pY783, Cell Signaling Technology, catalog 2821), and phospho–ZAP-70 (pY319, Cell Signaling Technology, catalog 2701) and developed using HRP-conjugated secondary antibodies (Amersham, GE Healthcare anti–mouse IgG HRP and anti–rabbit IgG HRP, catalogs NA931 and NA934, respectively) and ECL reagent (SuperSignal West Pico Plus ECL substrate, Thermo Fisher Scientific). Membranes were stripped using Restore Western Blot Stripping buffer (Thermo Fisher Scientific, catalogs 4958, 5690 and 2709) and reprobed using antibodies specific for total SLP-76, PLCγ, and ZAP-70 (Cell Signaling Technology). Chemiluminescence was detected and quantified with a Chemidoc Imager and Image Lab software (Bio-Rad).

### Flow cytometry and ImmTAAI–target cell binding studies.

Flow cytometric studies of human cell lines and primary T cells were conducted using the following fluorophore-conjugated antibodies: anti-CD3 (Invitrogen, clone OKT3), anti-CD4 (Invitrogen, clone RPA-T4), anti-CD8 (Invitrogen, clone RPA-T8), anti–PD-1 (BioLegend, clone EH12.2H7), anti–HLA-A2 (BioLegend, clone BB7.2), anti–PD-L1 (BioLegend, clone 29E.2A3), anti-CD80 (BD Pharmingen, clone L307.4), anti-CD86 (BD Pharmingen, clone FUN-1). After staining for dead cells with Live/Dead Fixable Aqua Dead Stain (Invitrogen), cell surface staining was conducted using 1 in 100 dilutions of the antibodies in FACS wash buffer (FWB; PBS, 0.5% FBS), incubating at 4°C for 30 minutes, followed by 2 washes in FWB. PPI_15–24_ HLA-A2–specific binding of FLAG-tagged ImmTAAI molecules to target cells was determined by incubating target cells (with or without prior PPI_15–24_ peptide pulsing) with ImmTAAI dilutions in growth media at 37°C, 5% CO_2_, for 3 hours. Target cells were washed in FWB and stained with an anti–FLAG-PE antibody (BioLegend, catalog 329706, clone L5) as described for the cell surface antibodies. Data were acquired on an IntelliCyt iQue Plus (Sartorius), and estimates of surface molecule/cell were calculated using PE Quantibrite Bead analysis (BD Biosciences). ImmTAAI binding dose response data were analyzed in Prism (GraphPad) using a 4 parameter, nonlinear least squares fit to calculate EC_50_ values

### Generation of T cell clones.

T cell clones specific for the pancreatic β cell antigen PPI_6–14_ (HLA-A*02:01 RLLPLLALL) were generated as described (61). T cell clone specificity was validated by dextramer staining (made in-house from biotinylated PPI_15–24_ pHLA-A2 and fluorescently labeled Streptavidin-Dextramer, Immundex ApS) and specific killing of EndoC-βH2-A2 Red cells pulsed with RLLPLLALL peptide (Incucyte S3, Sartorius).

### T cell killing assay and cytokine analyzes.

EndoC-βH2 Red cells were generated by transducing EndoC-βH2 cells with HLA-A2 β2-microglobulin and NucLight red (Sartorius). Target cells were plated at 5 × 10^4^ cells per well of a 96-well plate in Optiβ3 media, incubated over night at 37°C, 5% CO_2_, and then pulsed with PPI_6–14_ peptide at the indicated concentrations for 2 hours. ImmTAAI molecules and controls were added at different concentrations and incubated for 2 hours. To initiate the assay, T cell clones were washed twice and added to EndoC-βH2 Red target cells at 5 × 10^4^ cells per well in T cell cloning media. PD-L1 transduced EndoC-βH2 Red target cells with or without anti–PD-L1 blocking antibody were used as additional controls. Cell killing was determined by quantification of EndoC-βH2 Red cell number over time using the IncuCyte S3 imaging system (Sartorius). The number of red nucleus-labeled cells at each time point was normalized to the initial number of objects to take into account variation in cell density in the area visualized. The number of events was acquired in 4 images and averaged. Cytokine release was measured by V-PLEX Plus Proinflammatory Panel 1 (human) kit in accordance with the manufacturer’s instructions (Meso Scale Diagnostics [MSD]) using culture supernatants from the IncuCyte killing assays at 24 hours after time point. For the cytokine assay, nonstimulated T cells alone were assessed as additional controls. Cytokine release was normalized against stimulated controls, and ImmTAAI dose response data were analyzed in Prism (GraphPad) using a 4-parameter, nonlinear least squares fit to determine IC_50_ values.

### SPR kinetic analysis.

SPR competition binding analysis was performed using a BIAcore 8K. Briefly, biotinylated PPI peptide HLA-A2 complexes were immobilized on a streptavidin-coated CM5 chip. ImmTAAI molecules were captured onto the chip via the affinity-enhanced PPI_15–24_ TCR–PPI_15–24_ peptide HLA-A2 interaction. Excess PD-1 was passed over the chip (1 μM or 10× *K_D_* of each PD-1 antibody), followed by an excess of PD-1 and PD-L1–Fc (15 μM) (Supplemental Figure 3A).

### Statistics.

Statistical analysis was performed using GraphPad Prism. Unless stated otherwise, data are presented as the mean ± SD. Data were analyzed using 2-way ANOVA with repeated measures by Tukey’s or Sidak’s multiple-comparison test or by 1-way ANOVA and Dunnett’s multiple-comparison test. Four-parameter nonlinear regression analysis was used to fit dose-response curves and calculate IC_50_ and EC_50_ values.

### Study approval.

The Oxford A Research Ethics Committee approved protocol 13/SC/0226 (Immunocore study protocol number IMCres02) was used to obtain written consent for all blood donations and was fully approved by the National Research Ethics Committee South Central.

## Author contributions

APC, GB, JK, LJB, RF, RT, KW, EH, SJH, KP, NMGS, LMW, VG, and CRR performed and analyzed experiments. HG, DEOK, RO, DXO, and CML provided reagents. APC, GB, RF, RT, SH, TMM, and PW designed and interpreted experiments. APC, MLM, and PW prepared the manuscript. All authors critically read the manuscript.

## Supplementary Material

Supplemental data

Supplemental video 1

Supplemental video 2

## Figures and Tables

**Table 1:**
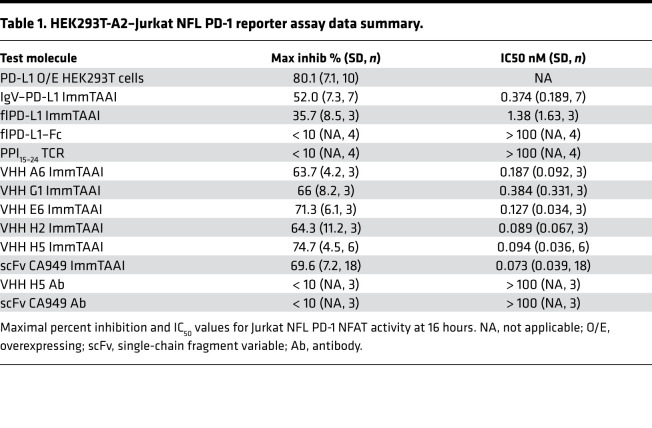
HEK293T-A2–Jurkat NFL PD-1 reporter assay data summary.

**Table 2:**
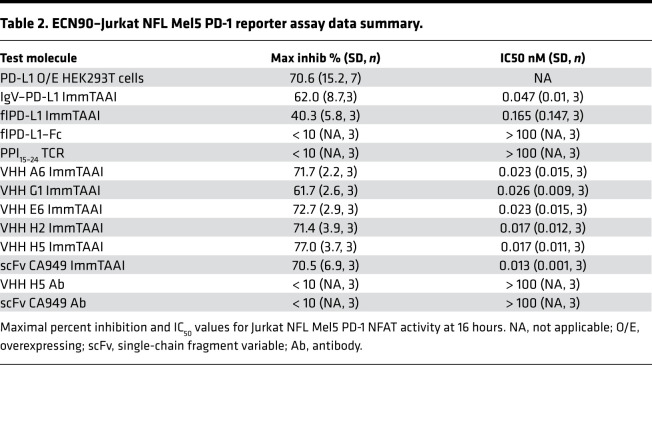
ECN90–Jurkat NFL Mel5 PD-1 reporter assay data summary.

**Table 3:**
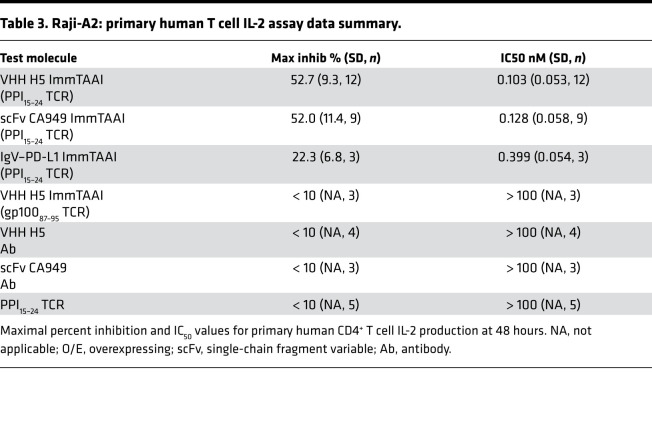
Raji-A2: primary human T cell IL-2 assay data summary.

**Table 4:**
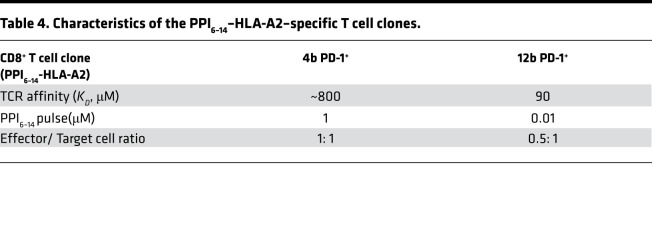
Characteristics of the PPI_6–14_–HLA-A2–specific T cell clones.

**Table 5:**
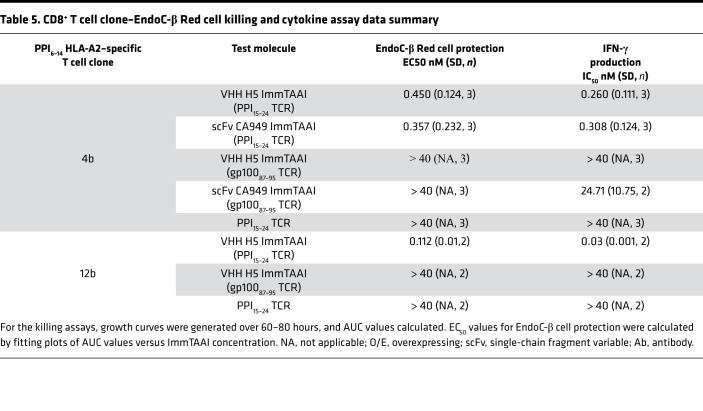
CD8^+^ T cell clone–EndoC-β Red cell killing and cytokine assay data summary

## Version 1 09/07/2021

In-Press Preview

## Version 2 10/22/2021

Electronic publication
