# Supplementary material for: Cell-targeted PD-1 agonists that mimic PD-L1 are potent T cell inhibitors
Source: JCI Insight. 2021 Oct 22;6(20):e152468. doi: 10.1172/jci.insight.152468 (PMC8564903; doi:10.1172/jci.insight.152468)
Supplement: Supplemental data [file jciinsight-6-152468-s224.pdf]

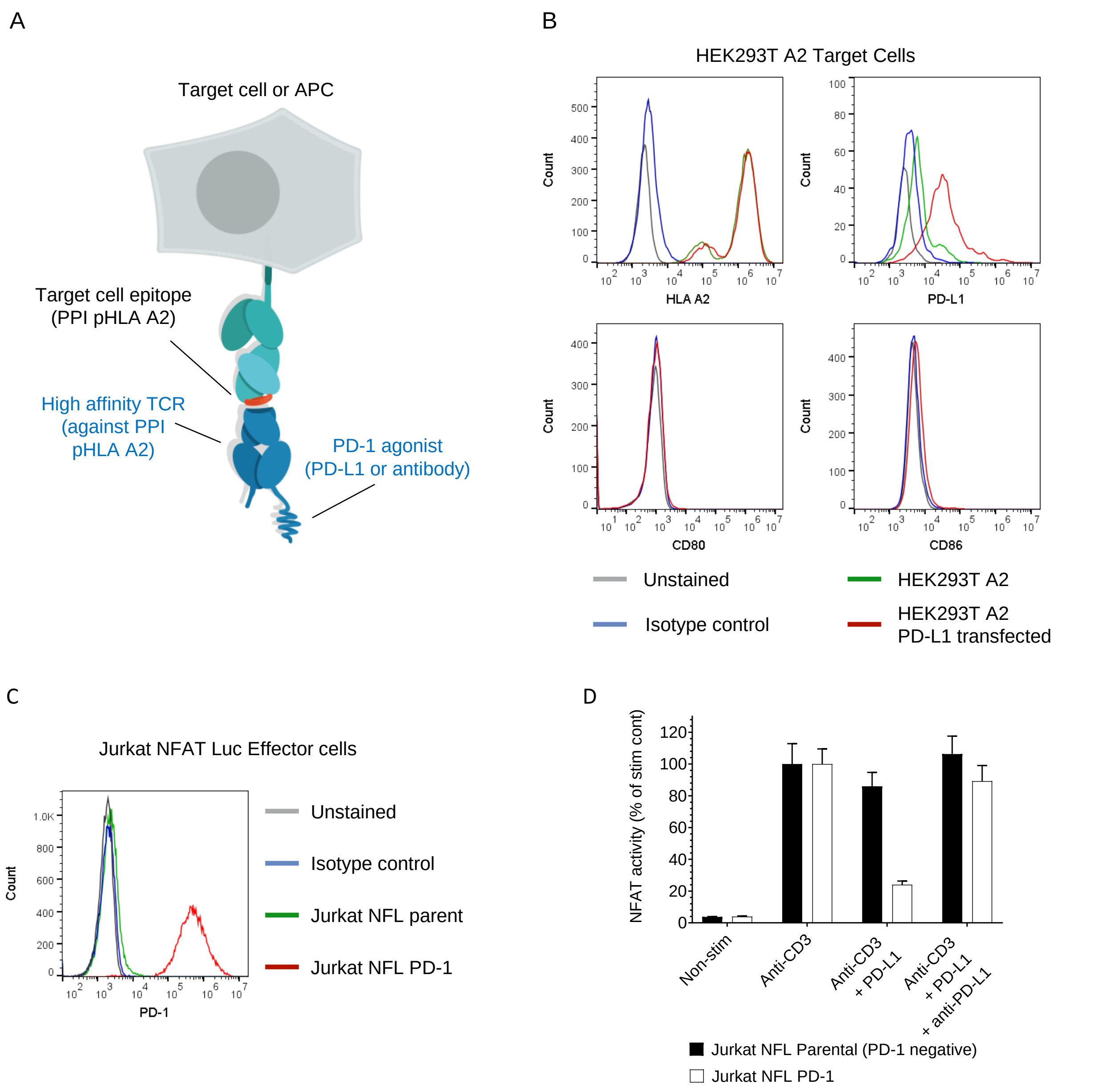

Supplementary Figure S1: Cell phenotyping and validation of the HEK293T A2: Jurkat PD-1 reporter assays. (A) Schematic of PD-1 agonist ImmTAAI **(B)** Flow cytometry analysis of HLA-A2, CD80, CD86 and PD-L1 expression in the reporter assay HEK293 A2 target cells +/- transfection with PD-L1 (representative histograms from 3 independent experiments) **C)** Flow cytometry analysis of PD-1 expression in Jurkat NFAT Luc cells +/- transfection with PD-1 (PD-L1 (representative histograms from 3 independent experiments). **(D)** Relative NFAT activity in Jurkat NFAT Luc cells +/- PD-1 co-incubated with HEK293T A2 target cells transfected with anti-CD3 antibody alone or anti-CD3 antibody plus PD-L1. HEK293T A2 target cells were plated out overnight and transfected with plasmids containing anti-CD3 antibody alone or anti-CD3 antibody plus PD-L1 for 24 hours. Where indicated, the transfected HEK293T A2 cells were pre-incubated with a blocking PD-L1 antibody for 30 minutes and Jurkat NFL or Jurkat NFL PD-1 effector cells were added at an E: T ratio of 1: 1. After 16 hours NFAT activity was determined by measuring luciferase luminescence and data normalised against TCR-stimulated controls in the absence of PD-L1. Data is plotted as mean ± SD (n = 3 and representative of 5 independent experiments).

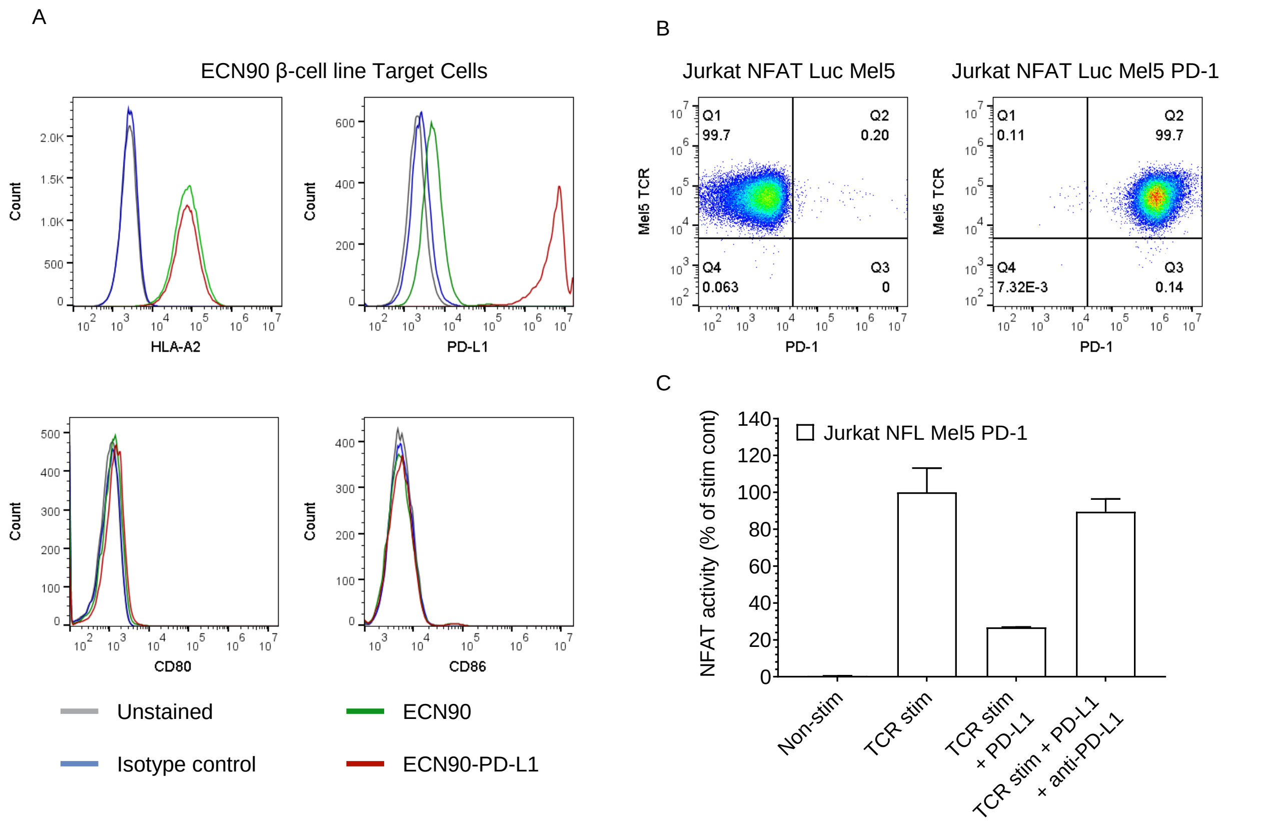

Supplementary Figure S2: Cell phenotyping and validation of the PD-1 ECN90: Jurkat NFL Mel5 PD-1 reporter assays. (A) Flow cytometry analysis of HLA-A2, CD80, CD86 and PD-L1 expression in the pancreatic β-cell line ECN90 +/- transfection with PD-L1 (representative histograms from 3 independent experiments). **B)** Flow cytometry analysis of PD-1 expression in Jurkat NFAT Luc Mel5 cells +/- transfection with PD-1 (representative histograms from 3 independent experiments). **(C)** Relative NFAT activity in Jurkat NFL Mel5 PD-1 effector cells co-incubated with ECN90 target cells +/- transfection with PD-L1. Parental or PD-L1 transduced ECN90 cells were plated overnight, pulsed with MelA activating peptide and, where indicated, pre-incubated with a blocking PD-L1 antibody for 30 minutes prior to adding Jurkat NFL Mel5 PD-1 effector cells. After 16 hours NFAT activity was measured as described above. Data is plotted as mean ± SD (n = 3 and representative of 3 independent experiments).

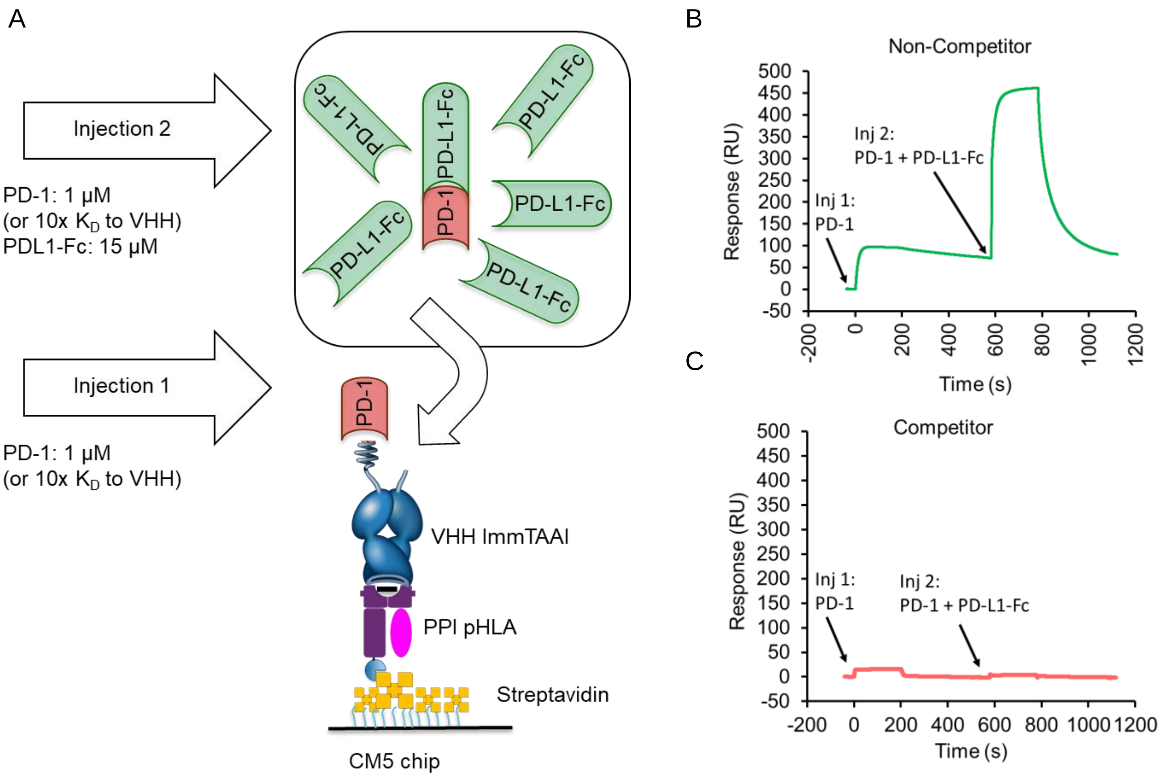

Supplementary Figure S3: Competition binding analysis of PD-1 antibody ImmTAAI and PD-L1 to PD-1. (A) Schematic depicting the method for SPR competition binding analysis of immobilized PD-1 antibody ImmTAAI molecules to soluble PD-1 in absence or presence of soluble PD-L1-Fc (see also Methods). **(B and C)** Representative sensorgram of a PD-L1 non-competitive PD-1 antibody ImmTAAI and a PD-L1 competitive PD-1 antibody ImmTAAI. Response units (RU) were plotted over time to characterize the binding of ImmTAAI molecules to PD-1 in the presence of PD-L1-Fc.

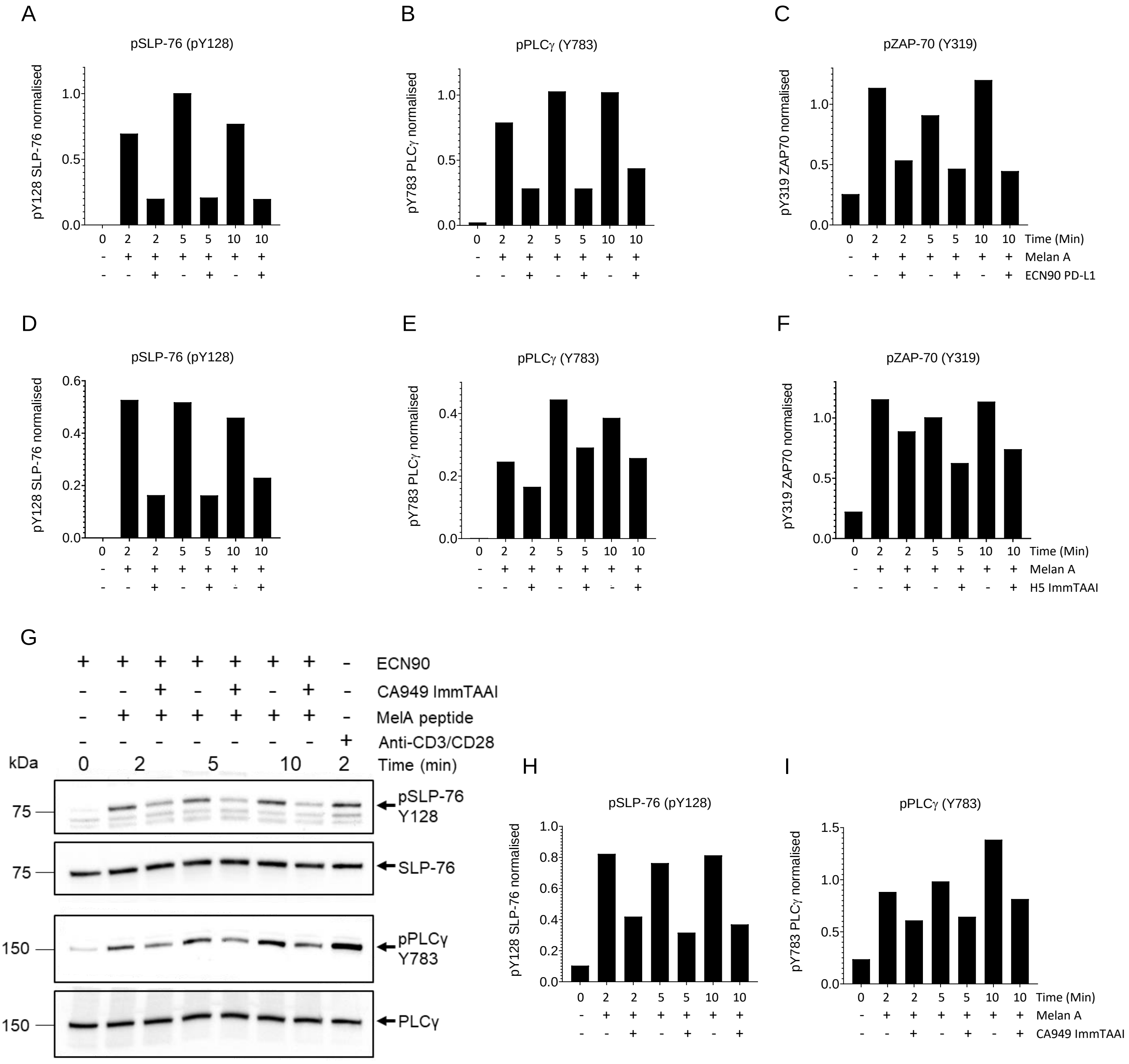

Supplementary Figure S4: PD-L1 expression, H5 and CA949 ImmTAAI molecules inhibit proximal TCR signalling. (A - C) Jurkat Mel5 PD-1 cells were stimulated with MelA-pulsed ECN90 or ECN90 PD-L1 cells and Western blotting performed as described in Figure 5. Chemiluminescent signals were quantified using ImageLab (BioRad). Histograms show the normalised densitometric data for phospho-SLP-76, phospho-PLCγ and phospho-ZAP-70 from the Western blots shown in Figure 5D (representative plots of 3 independent experiments). **(D - F)** Jurkat Mel5 PD-1 cells were stimulated with MelA-pulsed ECN90 cells, in the presence or absence of H5 ImmTAAI. Western blotting and signal quantification was done as described above. Histograms show the normalised densitometric data from the Western blots shown in Figure 5E (representative plots of 3 independent experiments). **(G)** Jurkat Mel5 PD-1 cells were stimulated with MelA-pulsed ECN90 cells, in the presence or absence of the scFv antibody CA949 ImmTAAI, for the indicated times. Western blotting was done as described above **(H and I)** histograms show the normalised densitometric data from the Western blots shown in Supplemental Figure 4G (representative plots of 3 independent experiments).

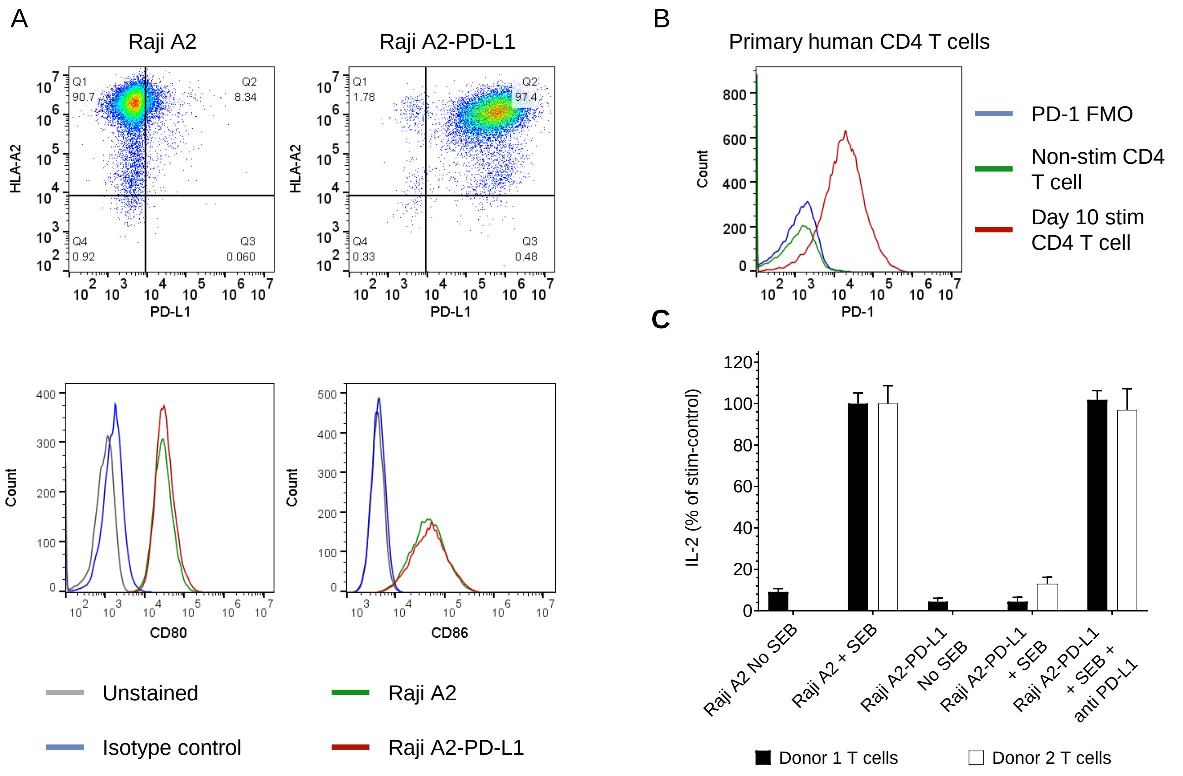

Supplementary Figure S5: Characterisation and validation of the Raji A2 : primary human CD4 T cell IL-2 assay. (A) Flow cytometry analysis of HLA-A2, CD80, CD86 and PD-L1 expression in Raji A2 and Raji A2 PD-L1 target cells (representative dot plots and histograms from 3 independent experiments). **(B)** Flow cytometry analysis of PD-1 expression in non-stimulated and day 10 Raji A2-SEB-stimulated primary human CD4 T cells (representative histograms from 3 independent experiments). **(C)** Impact of Raji A2 cells +/- PD-L1 transfection on IL-2 secretion using T cells from two donors. T cells were isolated from PBMC and were pre-activated by incubating with SEB-loaded and irradiated Raji A2 cells for 10 days. For re-stimulations, fresh SEB-loaded Raji A2 or Raji A2 PD-L1 cells were prepared and preincubated with or without a blocking PD-L1 antibody for 30 minutes. Pre-activated T cells were washed, rested for 4 hours and added to the SEB-loaded Raji A2 cells as indicated. After 48 hours supernatants were collected and IL-2 levels measured by ELISA. IL-2 release for each sample was normalized to stimulated control Data is plotted as mean ± SD (n = 4 and representative of 7 independent experiments).

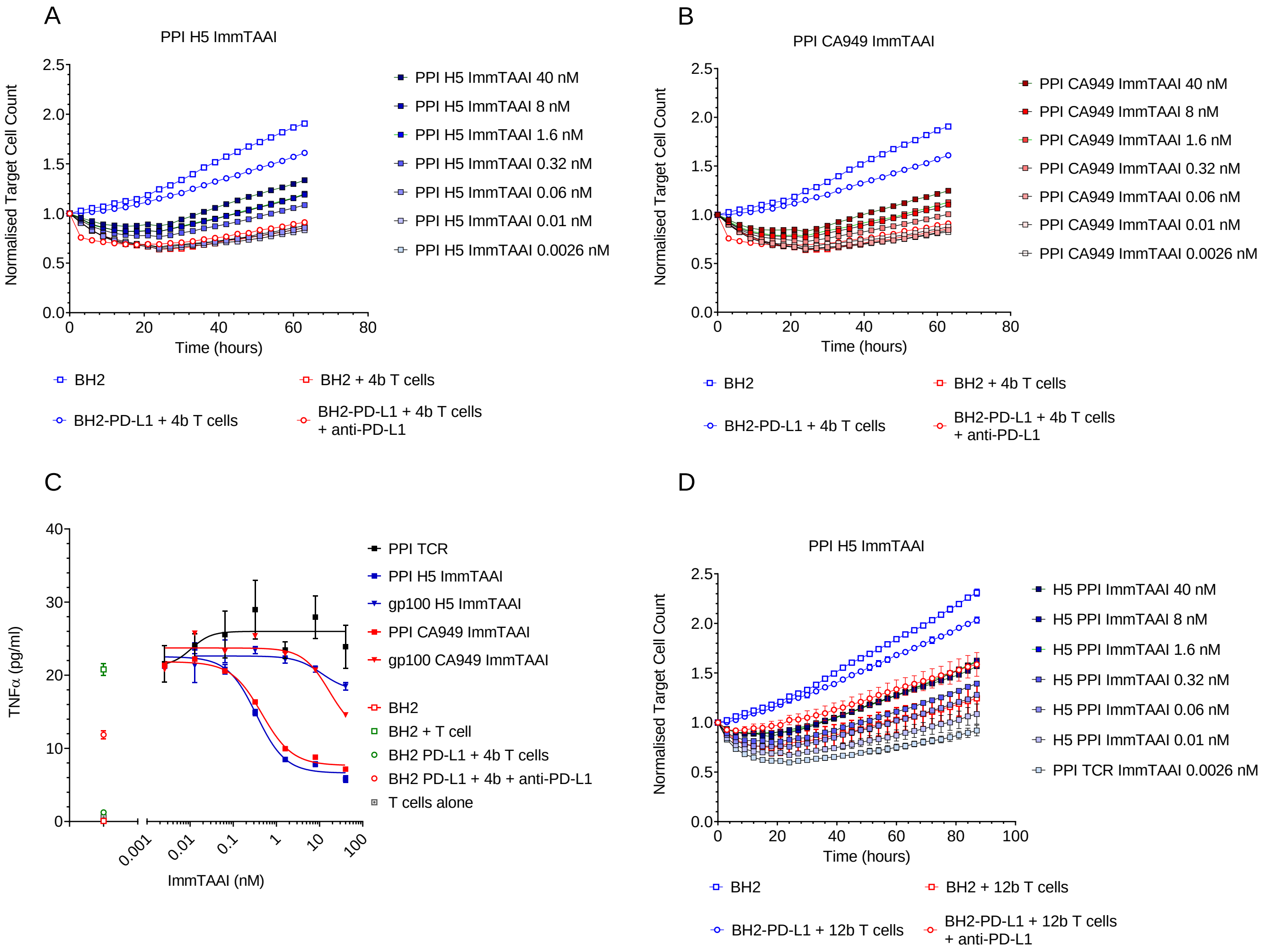

Supplementary Figure S6: PD-1 antibody ImmTAAIs inhibit target cell killing and inflammatory cytokine production by autoreactive T cell clones. Target cells (EndoC-β Red cells and EndoC-β Red PD-L1$^+$ cells) were plated into a 96 well plate in Optiβ3 media and incubated over night at 37°C/5% CO_2. Target cells were pulsed with $PPI_{6\text{-}14}$ peptide and PD-1 agonist antibody ImmTAAIs titrations were added and incubated for 2 hours. T cell clones were added to target cells and the number of red nucleus-labelled cells were quantified over time by the IncuCyte S3 imaging system and growth curves generated. **(A and B)** Growth curves of EndoC-β Red target cells co-cultured with clone 4b-PD-1$^+$ T cells alone or with titrations of the indicated PD-1 agonist antibody ImmTAAIs (representative plots of 3 independent experiments). **(C)** Dose-dependent inhibition of clone 4b-PD-1$^+$ TNFα production by H5 and CA949 PPI ImmTAAIs (n = 2, representative plots of 3 independent experiments). **(D)** Growth curves of EndoC-β Red target cells co-cultured with clone 12b-PD-1$^+$ T cells alone or with titrations of the indicated H5 antibody ImmTAAIs (representative plots of 3 independent experiments).

Supplementary Video 1: EndoC-β Red target cells co-cultured with clone 4b-PD-1+ T cells and imaged on an Incucyte S3 Imager for 4 days.

Supplementary Video 2: EndoC-β Red target cells co-cultured with clone 4b-PD-1+ T cells in the presence of 1.6 nM H5 ImmTAAI and imaged on an Incucyte S3 Imager for 4 days
